# How Bases Catalyze Diels‐Alder Reactions

**DOI:** 10.1002/chem.202203121

**Published:** 2022-12-13

**Authors:** Song Yu, Eveline H. Tiekink, Pascal Vermeeren, F. Matthias Bickelhaupt, Trevor A. Hamlin

**Affiliations:** ^1^ Department of Theoretical Chemistry Amsterdam Institute of Molecular and Life Sciences (AIMMS) Amsterdam Center for Multiscale Modeling (ACMM) Vrije Universiteit Amsterdam De Boelelaan 1083 1081 HV Amsterdam (TheNetherlands; ^2^ Institute for Molecules and Materials (IMM) Radboud University Heyendaalseweg 135 6525 AJ Nijmegen (The Netherlands; ^3^ Department of Chemical Sciences University of Johannesburg Auckland Park Johannesburg 2006 South Africa

**Keywords:** activation strain model, base catalysis, density functional calculations, Diels-Alder reactions, reactivity

## Abstract

We have quantum chemically studied the base‐catalyzed Diels‐Alder (DA) reaction between 3‐hydroxy‐2‐pyrone and N‐methylmaleimide using dispersion‐corrected density functional theory. The uncatalyzed reaction is slow and is preceded by the extrusion of CO_2_ via a retro‐DA reaction. Base catalysis, for example, by triethylamine, lowers the reaction barrier up to 10 kcal mol^−1^, causing the reaction to proceed smoothly at low temperature, which quenches the expulsion of CO_2_, yielding efficient access to polyoxygenated natural compounds. Our activation strain analyses reveal that the base accelerates the DA reaction via two distinct electronic mechanisms: i) by the *HOMO‐raising* effect, which enhances the normal electron demand orbital interaction; and ii) by donating charge into 3‐hydroxy‐2‐pyrone which accumulates in its reactive region and promotes strongly stabilizing secondary electrostatic interactions with N‐methylmaleimide.

## Graphical abstract

**All about that base**! Our quantum chemical activation strain analyses reveal how bases can effectively catalyze the Diels‐Alder reaction by promoting both the normal electron demand orbital interactions and electrostatic interactions. This study demonstrates the underlying mechanistic differences between the base‐catalyzed and Lewis acid‐catalyzed Diels‐Alder reactions.

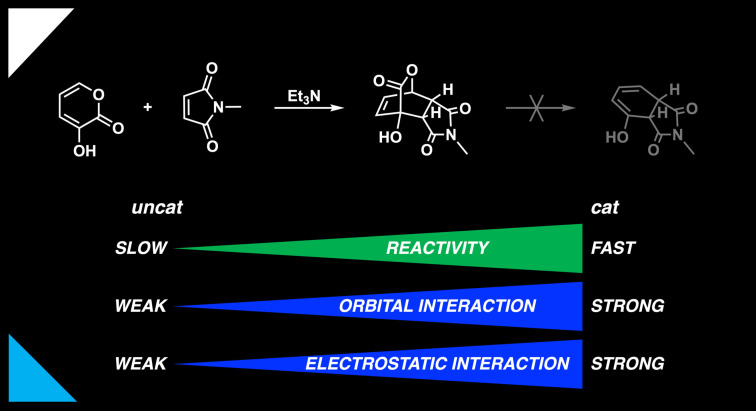

## Introduction

**Scheme 1:**
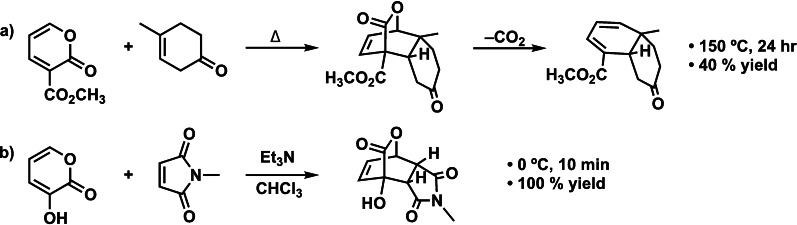
a) Uncatalyzed Diels‐Alder reaction of 2‐pyrone and b) base‐catalyzed Diels‐Alder reaction of 3‐hydroxy‐2‐pyrone.

Base‐catalyzed Diels‐Alder reactions are vital in organic synthesis.[^1^, ^2^] One typical example is the base‐catalyzed Diels‐Alder reaction between 2‐pyrone, acting as a diene, and a dienophile.^[1]^ The analogous uncatalyzed Diels‐Alder reaction of 2‐pyrone was first described by O. Diels and K. Alder in 1931^[3a]^ and later found wide applications in the synthesis of complex molecules and natural products.[^3b^, ^3c^] This uncatalyzed reaction is found to be slow, even at a high temperature, and is generally followed by a retro‐Diels‐Alder reaction expulsing CO_2_ (Scheme 1a).^[4]^ In 1995, Okarnura et al. improved the Diels‐Alder reaction of 2‐pyrone by using a base as a catalyst.^[1a]^ They revealed that the Diels‐Alder reaction between 3‐hydroxy‐2‐pyrone and N‐methylmaleimide catalyzed by triethylamine (Et_3_N) achieved a 100 % yield in 10 minutes at 0 °C (Scheme 1b), while its uncatalyzed analog had not been completed after 12 h at room temperature.^[1a]^ Interestingly, the base‐catalyzed reaction was not accompanied by the retro‐Diels‐Alder reaction at such a low temperature, making it a useful strategy for synthesizing polyoxygenated natural products.^[1]^ 3‐Hydroxy‐2‐pyridone and several electron‐deficient dienophiles have been found suitable for this reaction.[^1a^, ^1b^, ^1c^] Chiral bases such as alkaloid cinchonine, on the other hand, have been utilized in base‐catalyzed asymmetric Diels‐Alder reactions.[^1d^, ^1e^, ^1f^, ^1g^, ^1h^, ^1i^, ^1j^, ^1k^, ^1l^, ^1m^]

**Scheme 2:**
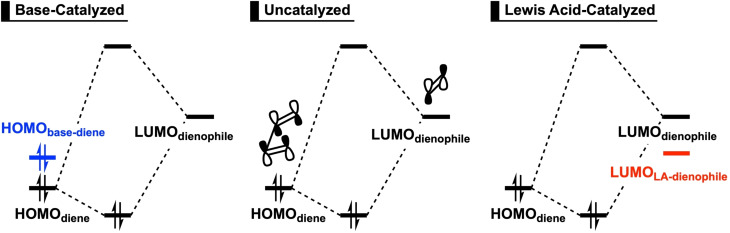
Schematic normal electron demand (NED) interaction diagrams for uncatalyzed and catalyzed Diels‐Alder reactions.

However, a thorough understanding of base‐catalyzed Diels‐Alder reactions is lacking. One can envisage a catalytic mechanism that hinges on enhancing the normal electron demand (NED) HOMO−LUMO orbital interaction whereby a catalytic amount of base (electron‐donating molecule) binds to the diene and destabilizes the HOMO (*HOMO‐raising*) (Scheme 2), leading to a reduced NED orbital‐energy gap and hence an enhanced HOMO−LUMO orbital interaction. This resembles the catalytic process proposed in earlier studies on Lewis acid (LA)‐catalyzed Diels‐Alder reactions:^[5]^ Complexation of a LA to the dienophile stabilizes the LUMO of the dienophile (*LUMO‐lowering*) and, therefore, reduces the NED orbital‐energy gap (Scheme 2), thereby enhancing the HOMO−LUMO orbital interaction. Recently, however, we have shown that this rationale behind the rate acceleration of LA‐catalyzed Diels‐Alder reactions is, in general, incorrect. We, in fact, found via our activation strain and Kohn‐Sham molecular orbital analyses that LAs promote the Diels‐Alder reaction by reducing the Pauli repulsion between the π‐systems of the diene and dienophile, and not due to the enhanced donor−acceptor interactions.^[6]^ In this work, we aim to expand the scope of our investigations and study base‐catalyzed Diels‐Alder reactions, with the goal of determining the underlying physical mechanism of the rate‐enhancement of base‐catalyzed Diels‐Alder reactions (Scheme 1).

**Scheme 3:**
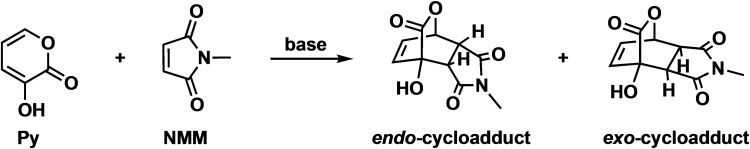
Base‐catalyzed Diels‐Alder reaction between **Py** and **NMM**.

To this end, we have investigated the archetypal base‐catalyzed Diels‐Alder reaction between 3‐hydroxy‐2‐pyrone (**Py**) and N‐methylmaleimide (**NMM**) (Scheme 3)^[1a]^ using dispersion‐corrected density functional theory (DFT) calculations at BLYP‐D3(BJ)/TZ2P. We concentrated on deciphering the catalytic effect of triethylamine (Et_3_N), which is typically used in experiment.^[1]^ For comparison, we have also evaluated the performance of other bases, such as the related base trimethylamine (Me_3_N) and the weaker base water (H_2_O). The activation strain model (ASM)^[7]^ with a matching canonical energy decomposition analysis (EDA) and quantitative molecular orbital theory^[8]^ were applied to reveal the physical factors behind the origin of the catalysis.

## Computational Methods

### Computational details

All calculations were performed with ADF2019.^[9]^ The geometries and energies were computed using dispersion‐corrected density functional theory at BLYP‐D3(BJ)/TZ2P. This approach comprises the GGA functional BLYP^[10]^ augmented by Grimme's D3 dispersion correction^[11]^ using the damping function proposed by Becke and Johnson.^[12]^ The basis set TZ2P is of triple *ζ* quality augmented with polarization functions.^[13]^ Our previous benchmark on the DFT method for various cycloaddition reactions has proven that this method performs excellently in calculating the trends in reactivity for cycloaddition reactions when weak interactions are involved in the systems.^[14]^ The accuracies of the fit scheme (Zlm fit)^[15a]^ and the integration grid (Becke grid)^[15b]^ were set to VERYGOOD. Frequency calculations were performed to characterize the nature of the stationary points where local minima presented real frequencies while transition structures had one imaginary frequency associated with the transition vector (the vibrational normal mode associated with the reaction and with a negative force constant). The potential energy surface (PES) was calculated using the intrinsic reaction coordinate (IRC) method^[16]^ and was further analyzed with the aid of the PyFrag 2019 program.^[17]^ The influence of chloroform, a common solvent used in experiment,^[1]^ was evaluated by the conductor‐like screening model (COSMO).^[18]^ All structures were visualized using CYLview.^[19]^

### Activation strain model and energy decomposition analysis

Quantitative analyses of the potential energy surfaces (PESs) associated with the studied reactions were obtained by means of the activation strain model (ASM) of reactivity.^[7]^ The PES, that is, Δ*E*(*ζ*), was decomposed into the strain energy, Δ*E*_strain_(*ζ*), and interaction energy, Δ*E*_int_(*ζ*) [Equation 1]:

(1) $$\Delta E\left(\zeta \right)=\Delta E{}_{\mathrm{strain}}\left(\zeta \right)+\Delta E{}_{\mathrm{int}}\left(\zeta \right)$$

The Δ*E*_strain_(*ζ*) is associated with the rigidity and the structural deformation of the reactants from their equilibrium structure to the geometry they adopt at the coordinate of *ζ* during the reaction. The Δ*E*_int_(*ζ*) is related to the electronic structure of the reactants and their spatial orientation and takes the mutual interactions between the deformed reactants into account.

To obtain a deeper insight into the physical mechanism behind Δ*E*_int_(*ζ*), we employed our canonical energy decomposition analysis (EDA),^[8]^ which decomposes the Δ*E*_int_ between the deformed reactants, within the framework of Kohn‐Sham DFT, into four physically meaningful terms [Equation 2]:

(2) $$\Delta E{}_{\mathrm{int}}\left(\zeta \right)=\Delta V{}_{\mathrm{elstat}}\left(\zeta \right)+\Delta E{}_{\mathrm{Pauli}}\left(\zeta \right)+\Delta E{}_{\mathrm{oi}}\left(\zeta \right)+\Delta E{}_{\mathrm{disp}}\left(\zeta \right)$$

The electrostatic interaction, Δ*V*_elstat_(*ζ*), corresponds to the classical electrostatic interaction between the unperturbed charge distributions of deformed reactants. The Pauli repulsion, Δ*E*_Pauli_(*ζ*), comprises the repulsion between closed‐shell orbitals and is, therefore, destabilizing. The orbital interaction, Δ*E*_oi_(*ζ*), accounts for the stabilizing orbital interactions, such as, charge transfer, namely, the interactions between the occupied orbitals of one reactant and the unoccupied orbitals of the other reactant, and polarization, that is, the occupied‐unoccupied orbital mixing within one reactant due to the presence of the other reactant. The dispersion term Δ*E*_disp_ corresponds to the dispersion corrections as introduced by Grimme et al.^[11]^

### Voronoi deformation density analysis

The electron density distribution is analyzed using the Voronoi deformation density (VDD) method^[20]^ for computing atomic charges. The VDD atomic charge on atom A (*Q*_A_^VDD^) is computed as the (numerical) integral of the deformation density in the volume of the Voronoi cell of atom A [Equation (3)]. The Voronoi cell of atom A is defined as the compartment of space bounded by the bond midplanes on and perpendicular to all bond axes between nucleus A and its neighboring nuclei.

(3) $$Q_{A}^{VDD}=-\underset{Voronoi\;cell\;of\;A}{\int^{\;}}\left[\rho \left(r\right)-\sum_{B}\rho_{B}\left(r\right)\right]dr$$

Here, *ρ*(**r**) is the electron density of the molecule, and ∑_*B*_ *ρ_B_* (**r**) the superposition of atomic densities *ρ_B_* of a fictitious promolecule without chemical interactions that is associated with the situation in which all atoms are neutral. The interpretation of the VDD charge *Q*_A_^VDD^ is rather straightforward and transparent: instead of measuring the amount of charge associated with a particular atom A, *Q*_A_^VDD^ directly monitors how much charge flows, due to chemical interactions, out of (*Q*_A_^VDD^>0) or into (*Q*_A_^VDD^<0) the Voronoi cell of atom A.

## Results and Discussion

### Base‐substrate complexation and trends in reactivity

First, we analyze the binding of various bases to the diene, 3‐hydroxy‐2‐pyrone (**Py**). The electronic and Gibbs free energies, relative to the infinitely separated base **B** and diene **Py**, of the base‐**Py** complex (**B‐Py**) and the infinitely separated protonated base cation [**B**+**H**]^**+**^ (**B** gains a proton and becomes a cation) with deprotonated **Py** anion [**Py**−**H**]^−^ (**Py** loses a proton and becomes an anion) were computed at BLYP‐D3(BJ)/TZ2P in the gas phase (Table 1) and in chloroform (Table S1). Table 1 shows that the **B‐Py** complex involves an interaction between the base and **Py**, which becomes increasingly more stabilizing from −10.4 to −12.5 to −13.0 kcal mol^−1^ for H_2_O to Et_3_N to Me_3_N, respectively. We find that the formal deprotonation of **Py** by the base ([**B**+**H]^+^** +[**Py**−**H**]^−^) is highly unlikely with respect to both the separated reactants and **B‐Py** complex. Thus, we can conclude that the **B‐Py** complex will be a likely intermediate in the base‐catalyzed Diels‐Alder reaction with N‐methylmaleimide (**NMM**).

**Table 1:** Electronic (Δ*E*, kcal mol^−1^) and Gibbs free energies (Δ*G*, kcal mol^−1^) associated with the formation of the **B**‐**Py** complex and the formal deprotonation of **Py** yielding [**B**+**H]^+^** +[**Py**−**H**]^−^.^[a]^

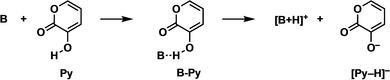				
**B**	**B‐Py**		**[B+H]^+^+[Py−H]^−^**	
	Δ*E*	Δ*G*	Δ*E*	Δ*G*
H_2_O	−10.4	0.2	121.8	129.4
Me_3_N	−13.0	−2.4	56.4	66.0
Et_3_N	−12.5	0.9	47.2	56.8

[a] All energies are relative to the infinitely separated base and **Py** computed at BLYP‐D3(BJ)/TZ2P.

Next, we employ the activation strain model (ASM) and energy decomposition analysis (EDA) to understand the nature of the interaction between the base and **Py** in the **B‐Py** complexes (Table 2). The strain energy (Δ*E*_strain_) associated with the formation of **B‐Py** complex becomes more destabilizing as the base goes from H_2_O to the stronger nitrogen bases Me_3_N and Et_3_N. Nevertheless, the interaction energy (Δ*E*_int_) is the primary contributor to the Δ*E*, and it becomes steadily more stabilizing from H_2_O to Me_3_N to Et_3_N, ranging from −13.0 to −18.1 kcal mol^−1^. For all bases, the electrostatic interaction (Δ*V*_elstat_) is the major contributor to the Δ*E*_int_ and shows, together with the dispersion energy (Δ*E*_disp_), the same trend as the interaction energies. The orbital interactions (Δ*E*_oi_), on the other hand, do roughly recover the trend in interaction energies in that they become more stabilizing from H_2_O to the nitrogen bases Et_3_N and Me_3_N. They are chiefly the result of the interaction between the donating lone‐pair orbital of the base and the accepting σ*_O−H_ orbital of **Py**. Note that Et_3_N is more basic than Me_3_N^[21]^ but has a slightly less favorable orbital interaction than Me_3_N upon complexation to **Py**. This is because the **Et_3_N‐Py** complex has a longer **B**⋅⋅⋅H−OR bond (1.68 Å) than **Me_3_N‐Py** (1.64 Å) induced by the sterically more encumbered ethyl groups, and therefore has a weaker orbital interaction than **Me_3_N‐Py**. Importantly, binding a base to **Py**, due to the above‐mentioned σ‐electron donation from the base to **Py**, leads to a more negative potential on the latter moiety and thus to a destabilization of its HOMO from −5.6 eV for HOMO_**Py**_ to −5.4 eV for HOMO_**H2O‐Py**_ to −4.9 eV for HOMO_**Et3N‐Py**_ and HOMO_**Me3N‐Py**_. This *HOMO‐raising* effect is, as we will show later, crucial for the rate‐enhancement of base‐catalyzed Diels‐Alder reactions.

**Table 2:** Activation strain and energy decomposition analysis terms (in kcal mol^−1^), O−H and **B**⋅⋅⋅H bond distances (in Å), and HOMO energies (eV) of **B**‐**Py** complexes.^[a,b]^

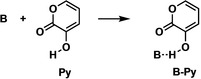										
**B**	Δ*E*	Δ*E*_strain_	Δ*E*_int_	Δ*V*_elstat_	Δ*E*_Pauli_	Δ*E*_oi_	Δ*E*_disp_	*r*_O−H_	*r*_B⋅⋅⋅H_	*ϵ*_HOMO_
None	–	–	–	–	–	–	–	0.99	–	−5.6
H_2_O	−10.4	2.6	−13.0	−24.9	31.3	−17.1	−2.3	1.00	1.72	−5.4
Me_3_N	−13.0	4.2	−17.2	−28.0	37.3	−21.2	−5.4	1.04	1.64	−4.9
Et_3_N	−12.5	5.6	−18.1	−28.8	40.0	−20.7	−8.5	1.04	1.68	−4.9

[a] The base and **Py** constitute the two interacting fragments. [b] Computed at BLYP‐D3(BJ)/TZ2P.

**Figure 1:**
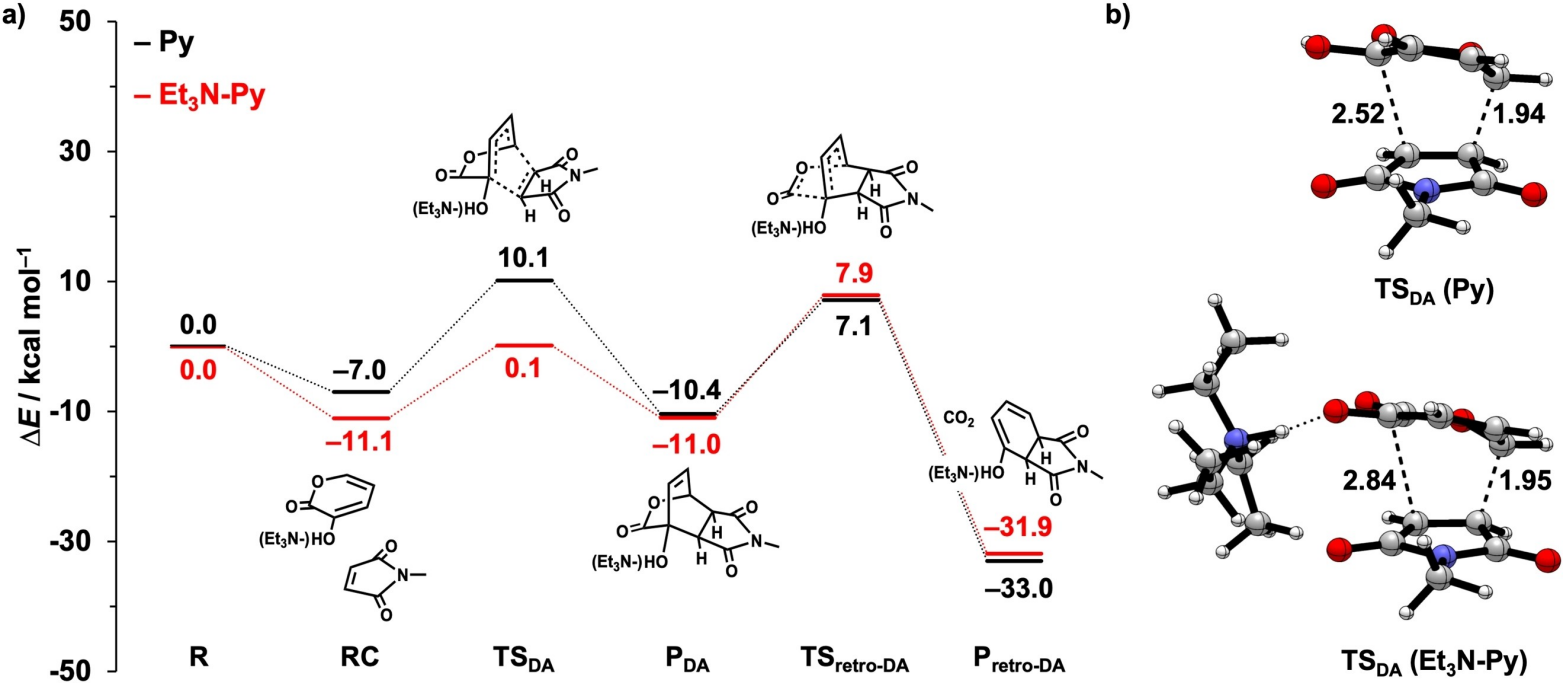
a) Computed energy profiles (Δ*E*, kcal mol^−1^) of the *endo‐*Diels‐Alder reactions of **Py** (black) and **Et_3_N**‐**Py** (red) with **NMM** and b) the corresponding geometries of TS_DA_ with newly forming bond lengths (Å). All were computed at BLYP‐D3(BJ)/TZ2P.

After analyzing the binding between the base and **Py**, we examine how the base catalyzes the Diels‐Alder (DA) reaction between the activated diene **B‐Py** and **NMM**. We focus on the uncatalyzed DA reaction and the Et_3_N‐catalyzed DA reaction, since Et_3_N is most frequently used to catalyze DA reactions in experiments.^[1a]^ The reaction profiles of other catalysts possess the same features and are provided in Figure S1 of the Supporting Information. The trends in reactivity on the electronic potential energy surface (PES) are the same as those on the Gibbs free energy PES (Figure S3) and those fully optimized and calculated in chloroform at COSMO(chloroform)‐BLYP‐D3(BJ)/TZ2P (Figures S4 and S5). Figure 1 shows the PES of the uncatalyzed and the Et_3_N‐catalyzed DA reaction between (**B‐)Py** and **NMM**, together with the transition state structures, following the *endo* pathway. The *exo* pathway shares the same reactivity trend but is, in accordance with experimental findings, disfavored relative to the *endo‐*pathways (Figures S2 and S8).[^1^, ^4^] The reactions proceed via the reactant complex (RC) and the DA transition state (TS_DA_) towards the cycloadduct (P_DA_), which, in turn, can undergo a retro‐DA reaction expulsing CO_2_ via a transition state (TS_retro‐DA_) to the final product (P_retro‐DA_). The uncatalyzed DA reaction (**Py**) has the highest activation barrier, with TS_DA_ at 10.1 kcal mol^−1^, and a concerted asynchronous reaction mode (Δ*r* ^TS^_C⋅⋅⋅C_ = 0.58 Å, where Δ*r* ^TS^_C⋅⋅⋅C_ refers to the difference in length between the newly forming C⋅⋅⋅C bonds in the transition state^[22]^). Binding the base Et_3_N to **Py** lowers the DA activation barrier by 10 kcal mol^−1^, to only 0.1 kcal mol^−1^, and increases the degree of asynchronicity of the reaction to Δ*r* ^TS^_C⋅⋅⋅C_ = 0.89 Å. In contrast, the base imparts only a small effect on the retro‐DA (*i. e*., dissociation of CO_2_) reaction barrier. With respect to the preceding DA cycloadduct P_DA_, the retro‐DA reaction barrier increases from 17.5 kcal mol^−1^ for the uncatalyzed to 18.9 kcal mol^−1^ for the base‐catalyzed reaction. Our computed PES nicely correlates with experimental observations where the uncatalyzed DA reaction between **Py** and **NMM** proceeds at an elevated temperature, which is needed to overcome the rate‐determining DA activation barrier and hence continues directly over the retro‐DA reaction which has a lower activation barrier (Scheme 1a).^[4]^ The Et_3_N‐catalyzed reaction, on the other hand, takes place at a low temperature owing to the relatively lower DA activation barrier, and can effectively be trapped prior to the kinetically unfavorable retro‐DA reaction, yielding a polyoxygenated product (Scheme 1b).^[1]^ If the Et_3_N‐catalyzed reaction would be performed at elevated temperatures, it is likely that the retro‐DA reaction would become feasible as in the case for the uncatalyzed reaction.

### Origin of base catalysis

**Figure 2:**
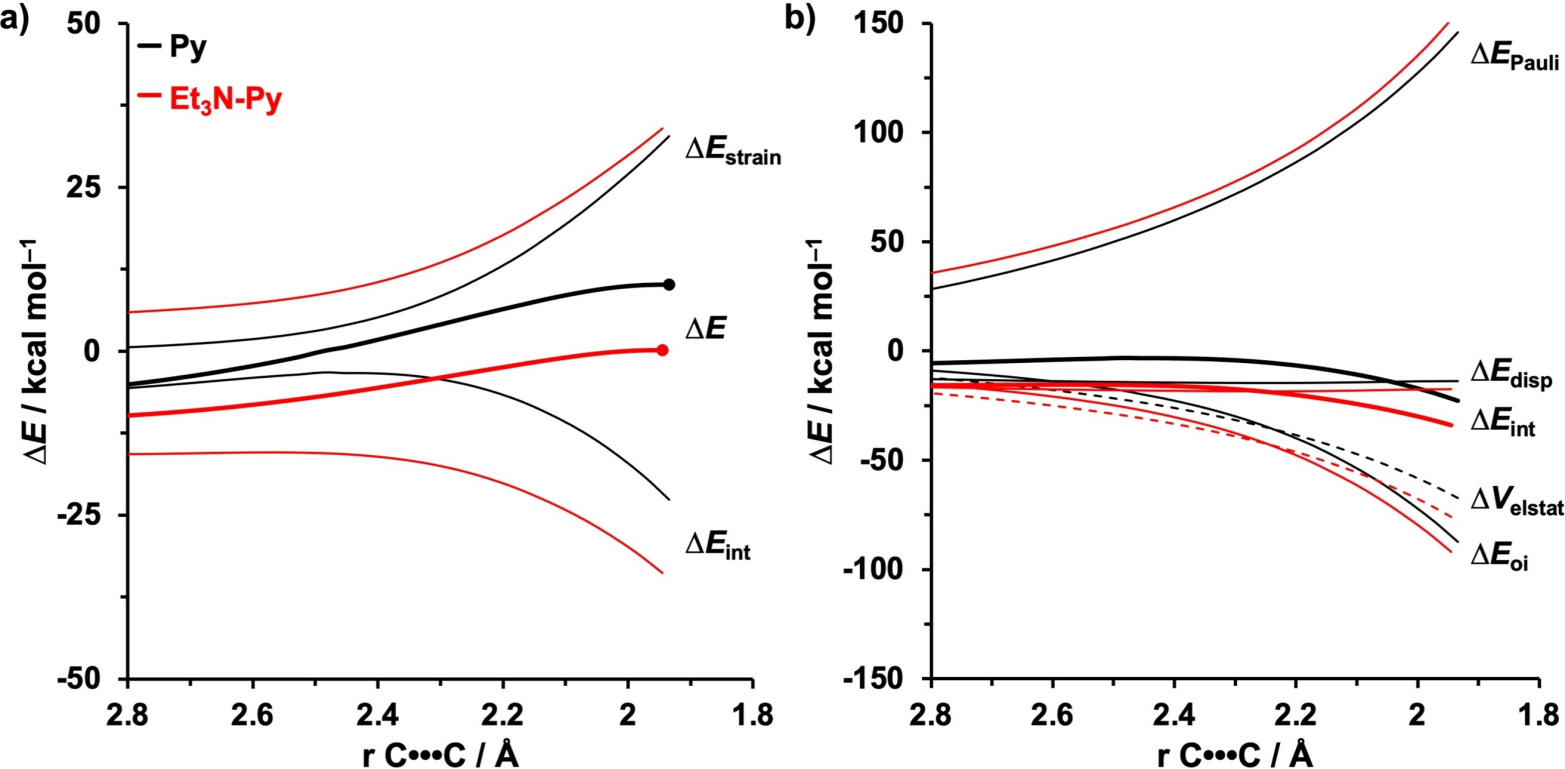
a) Activation strain and b) energy decomposition analyses of the *endo‐*Diels‐Alder reactions of **Py** (black) and **Et_3_N**‐**Py** (red) with **NMM** along the IRC projected on the shorter of the two newly forming C⋅⋅⋅C bonds (TSs indicated with a dot). Computed at BLYP‐D3(BJ)/TZ2P.

The physical factors leading to the enhanced reactivity of the base‐catalyzed compared to the uncatalyzed DA reaction are examined by applying the activation strain model (ASM) of reactivity,^[7]^ where the interacting fragments are the diene, (**B**‐**)Py**, and the dienophile, **NMM**. In both the activation strain and energy decomposition analysis diagrams, the intrinsic reaction coordinate (IRC) is projected onto the shorter of two newly forming C⋅⋅⋅C bonds between (**B**‐**)Py** and **NMM**. This critical reaction coordinate undergoes a well‐defined change during the reaction from the reactant complex via the transition state to the cycloadduct and has been shown to be a valid reaction coordinate for studying cycloadditions.[^6^, ^14^, ^23^] Figure 2a shows the activation strain diagrams from the reactants to the transition states for the uncatalyzed and Et_3_N‐catalyzed DA reactions (see Figures S6 and S7 for all transition state structures and analyses). The accelerated reactivity of the Et_3_N‐catalyzed reaction originates exclusively from a more stabilizing interaction energy. The strain energy, on the other hand, follows a trend that is opposite to the activation barriers and is, therefore, not responsible for the observed rate enhancement. To pinpoint the origin of the more stabilizing interaction energy, we applied the energy decomposition analysis (EDA) (Figure 2b).^[8]^ We find that both the orbital and electrostatic interactions are responsible for the observed trend in interaction energy because both these energy terms are more stabilizing for **Et_3_N**‐**Py** compared to **Py**. The Pauli repulsion shows an opposite trend, *i. e*., the Et_3_N‐catalyzed DA reaction goes with more destabilizing Pauli repulsion than the uncatalyzed analog and, hence, is not responsible for the observed reactivity trend. These findings demonstrate the difference in the underlying electronic mechanism between base‐catalyzed Diels‐Alder reactions (*HOMO‐raising* catalysis) and Lewis acid‐catalyzed Diels‐Alder reactions (*Pauli‐lowering* catalysis).

Next, we address why the Et_3_N‐catalyzed DA reaction experiences a more stabilizing orbital interaction compared to the uncatalyzed analog. In line with the textbook rationale behind base‐catalysis,[^2h^, ^2i^, ^2j^] the binding of Et_3_N to **Py** destabilizes the orbitals of **Py** and consequently strengthens the normal electron demand (NED) interaction, *i. e*., the donor−acceptor interaction between the filled molecular orbitals of (**B**‐)**Py** with virtual molecular orbitals of **NMM**. This effect, however, gets partly compensated, as Et_3_N simultaneously weakens the inverse electron demand (IED) interaction, *i. e*., the donor−acceptor interaction between the virtual molecular orbitals of (**B**‐)**Py** with filled molecular orbitals of **NMM**. By performing a Kohn‐Sham molecular orbital (KS‐MO)[^8b^, ^24^] analysis on the consistent geometries where the shorter forming bond between (**B**‐**)Py** and **NMM** is 1.95 Å,^[25]^ we find that the NED interaction energy becomes more stabilizing, going from −46.5 kcal mol^−1^ for the uncatalyzed to −58.5 kcal mol^−1^ for the Et_3_N‐catalyzed reaction (ΔΔ*E*_oi_^NED^=−12 kcal mol^−1^), while the IED interaction energy gets less stabilizing and goes from −35.1 kcal mol^−1^ for the uncatalyzed to −28.0 kcal mol^−1^ for the catalyzed case (ΔΔ*E*_oi_^IED^=7.1 kcal mol^−1^). Note that the strength of the NED interaction is obtained by performing EDA computations while having artificially removed all virtual orbitals on (**B**‐)**Py**, and the IED interaction is obtained by performing EDA computations while having artificially removed all virtual orbitals on **NMM**. It is apparent that the loss of IED interaction is small and cannot completely counteract the gain in NED interaction.

**Figure 3:**
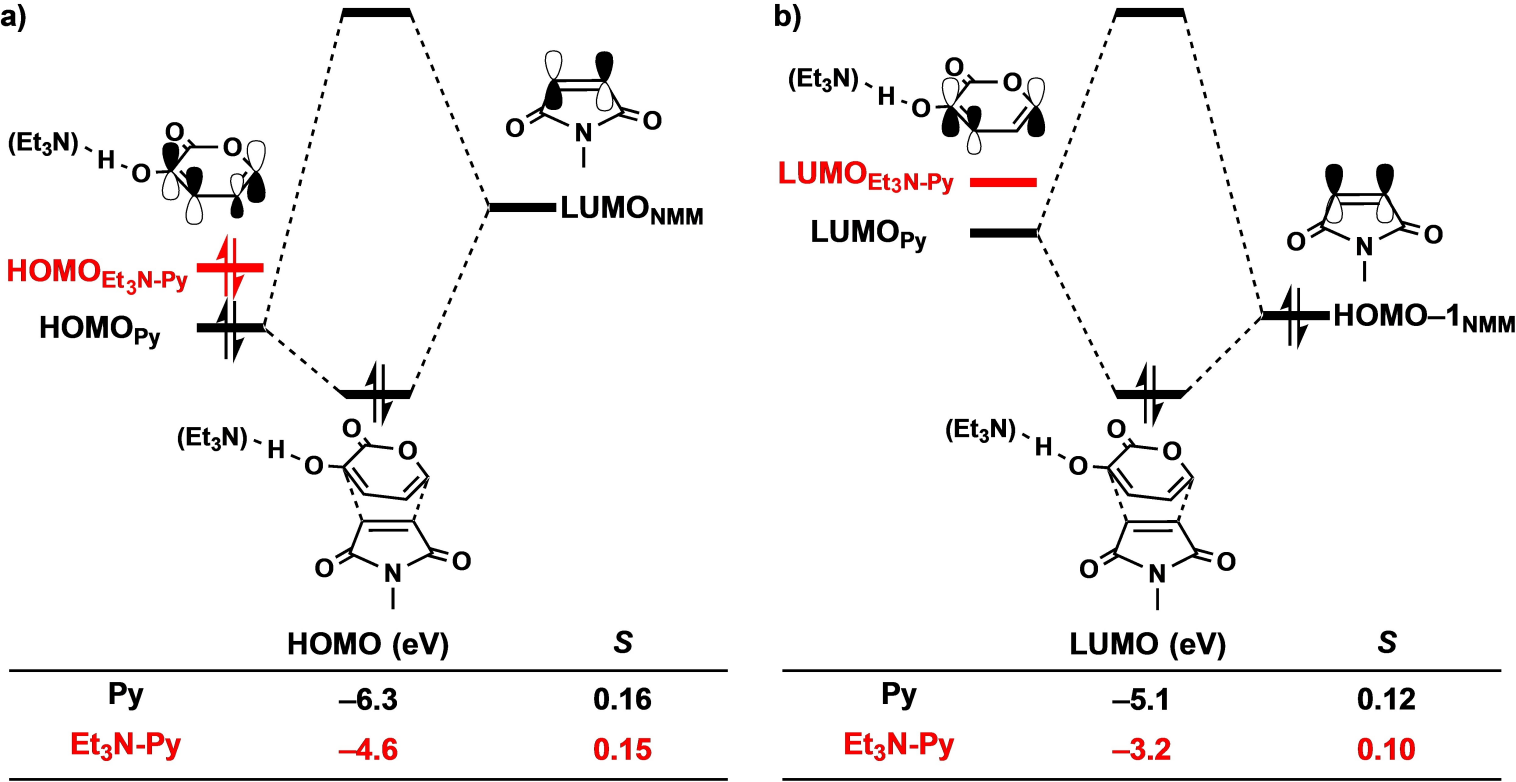
Schematic molecular orbital diagrams with key orbital interactions for a) the normal electron demand (NED) HOMO_(**B‐**)**Py**_−LUMO_**NMM**_ interaction and b) the inverse electron demand (IED) LUMO_(**B**‐)**Py**_−HOMO−1_**NMM**_ interaction of the uncatalyzed and Et_3_N‐catalyzed Diels‐Alder reaction between (**B**‐**)Py** and **NMM**, computed at consistent TS‐like geometries where the shorter of the newly forming C⋅⋅⋅C bonds between (**B**‐**)Py** and **NMM** is 1.95 Å, at BLYP‐D3(BJ)/TZ2P.

Moreover, we inspect the key NED interaction of HOMO_(**B**‐)**Py**_−LUMO_**NMM**_ and find that the strengthening of the NED interaction for the Et_3_N‐catalyzed reaction comes exclusively from a raised HOMO_(**B**‐)**Py**_. The HOMO_(**B**‐)**Py**_ is raised in energy from −6.3 eV for the uncatalyzed to −4.6 eV for the catalyzed reaction, leading to a smaller and thus more favorable orbital energy gap (Figure 3a). An inspection of the key IED interaction of LUMO_(**B**‐)**Py**_−HOMO−1_**NMM**_ shows that the weakening of the IED interaction along with the Et_3_N‐catalyzed reaction comes from a smaller energy gap induced by a destabilized LUMO_(**B‐**)**Py**_ with a reduced overlap (Figure 3b). Thus, we conclude that the enhanced orbital interactions associated with the Et_3_N‐catalyzed DA reaction between **Py** and **NMM** is solely the result of the *HOMO‐raising* effect of the base.

**Figure 4:**
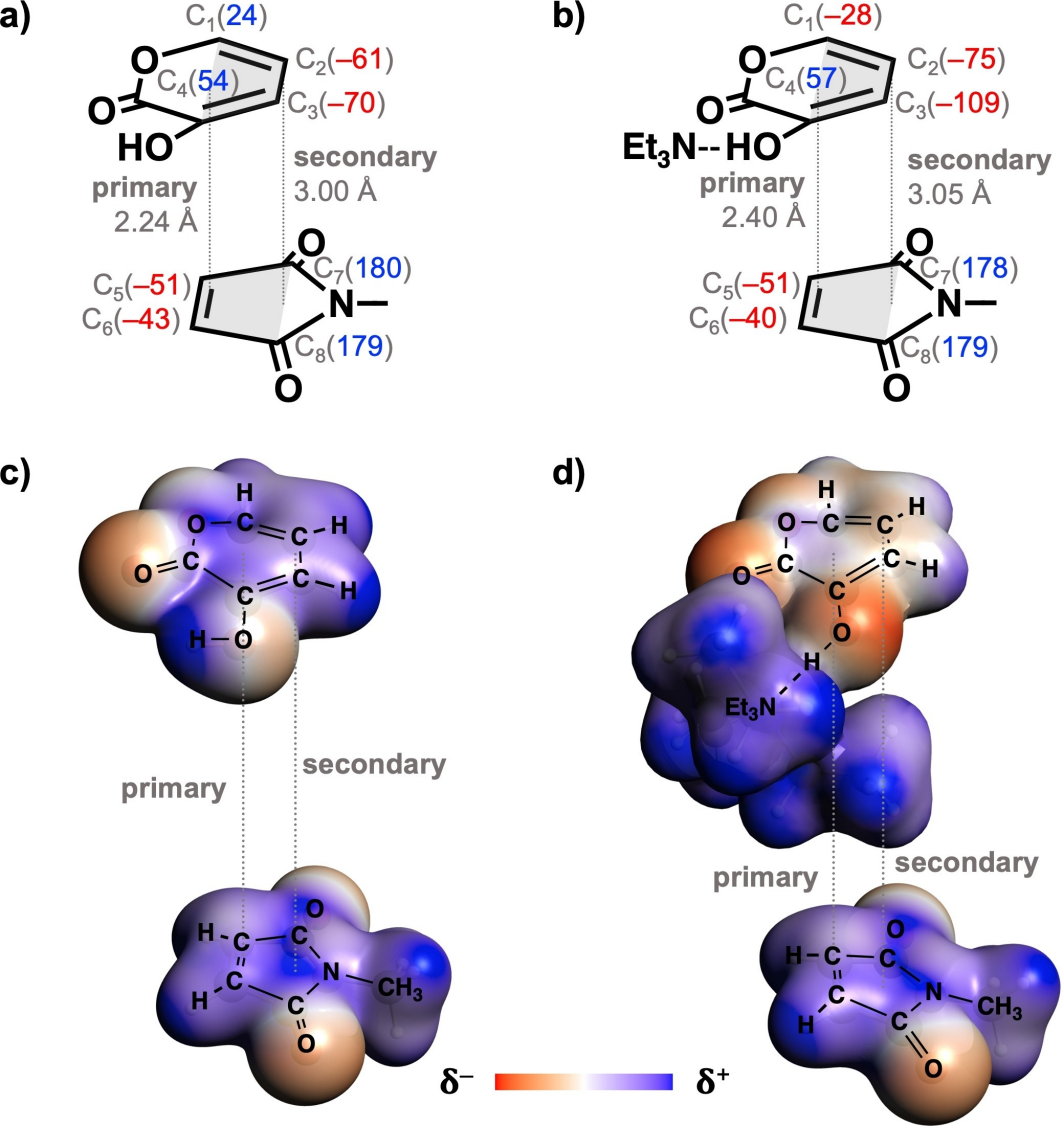
Schemes of electrostatic interactions in the Diels‐Alder reactions of a) **Py** and b) **Et_3_N**‐**Py** with **NMM** including the VDD charges (in milli‐electrons) at key atoms of individual reactants. MEPs (at 0.01 Bohr^−3^) from −0.09 to 0.09 Hartree of individual reactants during the Diels‐Alder reactions of c) **Py** and d) **Et_3_N**‐**Py** with **NMM**, computed at consistent TS‐like geometries where the shorter of the newly forming C⋅⋅⋅C bonds is 1.95 Å at BLYP‐D3(BJ)/TZ2P.

To understand the origin of the more stabilizing electrostatic interactions for the Et_3_N‐catalyzed DA reaction (Figure 2b), we analyze how, in **B**‐**Py**, coordinating the base to the diene **Py** has changed the charge density distribution of the **Py** moiety, using the Voronoi deformation density (VDD) method^[21]^ and the molecular electrostatic potential (MEP). This has been done for the uncoordinated reactant **Py** and for **B**‐**Py**, each at consistent TS‐like geometries. The latter are defined as the structures near the saddle‐point in which, for both reactions, the shorter of the newly forming C⋅⋅⋅C bonds between (**B**‐**)Py** and **NMM** assumes the exact same value of 1.95 Å. Our analyses reveal that the enhanced stabilization of the electrostatic interactions for the Et_3_N‐catalyzed DA reaction originates from the promoted secondary electrostatic interaction between the interacting reactants. During the DA reaction, (**B**‐**)Py** and **NMM** can engage in two attractive electrostatic interactions:^[26]^ i) the primary electrostatic interaction between the bond‐forming atoms, that is, of C_1_ and C_4_ in (**B**‐**)Py** with C_5_ and C_6_ of **NMM**, respectively; and ii) the secondary electrostatic attraction between C_2_ and C_3_ in (**B**‐**)Py** with C_7_ and C_8_ in **NMM**. For the uncatalyzed DA reaction between **Py** and **NMM**, the positively charged C_1_ and C_4_ atoms and the negatively charged C_2_ and C_3_ atoms of **Py** have a stabilizing electrostatic interaction with the negatively charged C_5_ and C_6_ atoms and the positively charged C_7_ and C_8_ atoms, respectively (Figure 4a and c). Binding Et_3_N to **Py** has a profound effect on the magnitude and distribution of the electron density of the **Py** moiety in **Et_3_N**‐**Py**, as electrons flow from Et_3_N to **Py** via the donor−acceptor interaction between the donating lone pair orbital of Et_3_N and the accepting σ*_H−O_ orbital of **Py** (Table 2). Accordingly, C_1_, C_2_, and C_3_ of **Et_3_N**‐**Py** become more negatively charged (Figure 4b and d). As a result, the secondary electrostatic interaction becomes significantly stronger, whereas the primary electrostatic interaction gets slightly weakened, ultimately yielding a more stabilizing electrostatic interaction for the Et_3_N‐catalyzed compared to the uncatalyzed DA reaction.

## Conclusion

Our computational study furnishes physical insight into the base‐catalyzed Diels‐Alder (DA) reaction between 3‐hydroxy‐2‐pyrone (**Py**) and N‐methylmaleimide (**NMM**). The uncatalyzed DA reaction is slow and is followed by a retro‐DA reaction expelling CO_2_. However, when catalyzed by a base, such as triethylamine, the DA activation barrier is lowered up to 10 kcal mol^−1^, causing the reaction to proceed smoothly at low temperature, thereby quenching the kinetically unfavorable expulsion of CO_2_. In this way, base catalysis affords efficient access to polyoxygenated cycloadducts at a low temperature.

Our quantum chemical analyses based on the activation strain model (ASM) and Kohn‐Sham molecular orbital theory identify that the base lowers the DA activation barrier through two mechanisms: i) by enhancing the normal electron demand (NED) interactions via the *HOMO‐raising* effect of the base; and ii) by stabilizing the secondary electrostatic interactions between the reactants. Complexation of a base to **Py** induces a strong electrostatic interaction and donor−acceptor interaction between the donating lone pair of the base and the σ_OH_* orbital of **Py**, resulting in a more negative potential on **Py**. The latter destabilizes the molecular orbitals of **Py**, in particular the HOMO of **Py**, and thus reduces the NED energy gap and enhances the stabilizing orbital interactions.

Finally, this work highlights the fundamentally different electronic mechanisms behind base‐catalyzed versus Lewis acid‐catalyzed DA reactions. Previously, we found that Lewis acids enhance the DA reactivity by reducing the Pauli repulsions between the π‐systems of the diene and dienophile, and not due to enhanced donor−acceptor orbital interactions.^[6]^ Taken altogether, we find that Lewis acids accelerate DA reactions via *Pauli‐lowering catalysis*, whereas bases accelerate DA reactions via *HOMO‐raising catalysis*.

## Conflict of interest

The authors declare no conflict of interest.

1

## Supporting information

As a service to our authors and readers, this journal provides supporting information supplied by the authors. Such materials are peer reviewed and may be re‐organized for online delivery, but are not copy‐edited or typeset. Technical support issues arising from supporting information (other than missing files) should be addressed to the authors.

Supporting Information

## Data Availability

The data that support the findings of this study are available in the supplementary material of this article.
