# Supplementary material for: How Bases Catalyze Diels‐Alder Reactions
Source: Chemistry. 2022 Dec 13;29(7):e202203121. doi: 10.1002/chem.202203121 (PMC10108159; doi:10.1002/chem.202203121)
Supplement: Supplementary file 1 — Supporting Information [file CHEM-29-0-s001.pdf]

Chemistry–A European Journal

Supporting Information

How Bases Catalyze Diels-Alder Reactions

Song Yu, Eveline H. Tiekink, Pascal Vermeeren, F. Matthias Bickelhaupt,* and Trevor A. Hamlin*

Contents

Table S1. Electronic ΔE and Gibbs free energies ΔG (in kcal mol^{-1}) of the **B-Py** complex and the formal deprotonation of **Py** yielding **[B+H]**$^+$ + **[Py–H]**$^-$. All energies are relative to the infinitely separated base and **Py**, computed at COSMO(chloroform)-BLYP-D3(BJ)/TZ2P.

Figure S1. a) Energy profiles ΔE for the *endo* Diels-Alder reactions of **Py** (black) and **H_2O-Py** (red) with **NMM**; and b) energy profiles ΔE for the *endo* Diels-Alder reactions of **Py** (black) and **Me_3N-Py** (red) with **NMM**. Computed at BLYP-D3(BJ)/TZ2P.

Figure S2. Energy profiles ΔE of the a) *endo*- and b) *exo*-Diels-Alder reactions of **Py** (black) and **Et_3N-Py** (red) with **NMM**. Computed at BLYP-D3(BJ)/TZ2P.

Figure S3. Free energy profiles ΔG of the a) *endo*- and b) *exo*-Diels-Alder reactions of **Py** (black) and **Et_3N-Py** (red) with **NMM**. Computed at BLYP-D3(BJ)/TZ2P.

Figure S4. Energy profiles ΔE of the a) *endo*-and b) *exo*-Diels-Alder reactions of **Py** (black) and **Et_3N-Py** (red) with **NMM**. Computed at COSMO(chloroform)-BLYP-D3(BJ)/TZ2P.

Figure S5. Free energy profiles ΔG of the a) *endo*-and b) *exo*-Diels-Alder reactions of **Py** (black) and **Et_3N-Py** (red) with **NMM**. Computed at COSMO(chloroform)-BLYP-D3(BJ)/TZ2P.

Figure S6. Transition states with the newly forming C···C bond lengths (Å), activation energies ($\Delta E^{\ddagger}$, kcal mol^{-1}), reaction energies (ΔE_{rxn}, kcal mol^{-1}), and length differences between the two forming bonds ($\Delta r^{TS}_{C\cdots C}$, Å), of the *endo*-Diels-Alder reactions between **(B-)Py** and **NMM**.

Figure S7. a) Activation strain and b) energy decomposition diagrams of *endo*-Diels-Alder reactions between **(B-)Py** and **NMM** along the IRC, projected on the shorter of the two newly forming C···C bonds (TS indicated by a dot). Computed at BLYP-D3(BJ)/TZ2P.

Figure S8. a) Activation strain and b) energy decomposition diagrams of *endo* and *exo*-Diels-Alder reactions between **Py** and **NMM**; c) activation strain and d) energy decomposition diagrams of *endo* and *exo*-Diels-Alder reactions between **Et_3N-Py** and **NMM**. All energy terms are projected on the shorter of the two newly forming C···C bonds (TS indicated by a dot). Computed at BLYP-D3(BJ)/TZ2P.

Table S2. Electronic energies, enthalpies, Gibbs free energies, the number and wavelength of the imaginary vibrational frequencies and cartesian coordinates, for all stationary points.

Table S1. Electronic Δ*E* and Gibbs free energies Δ*G* (in kcal mol^{-1}) of the **B-Py** complex and the formal deprotonation of **Py** yielding [**B**+**H**]$^{+}$ + [**Py**–**H**]$^{-}$. All energies are relative to the infinitely separated base and **Py**, computed at COSMO(chloroform)-BLYP-D3(BJ)/TZ2P.

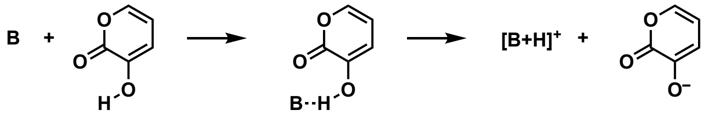

**B**	**B-Py**		**[B+H]$^{+}$ + [Py–H]$^{-}$**	
	Δ*E*	Δ*G*	Δ*E*	Δ*G*
H_2O	–6.9	3.6	70.1	69.6
Me_3N	–10.8	0.6	24.4	25.4
Et_3N	–10.3	1.6	21.6	22.1

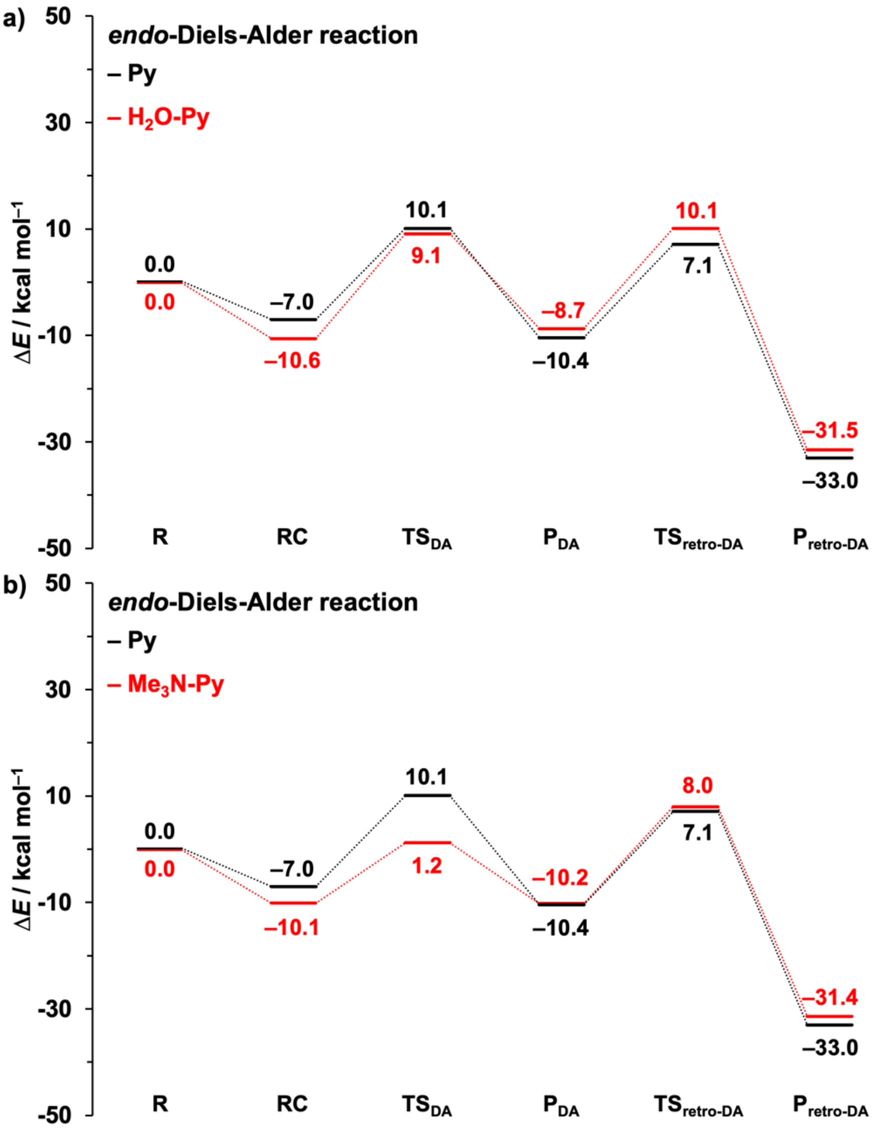

Figure S1. a) Energy profiles Δ*E* for the *endo* Diels-Alder reactions of **Py** (black) and **H_2O-Py** (red) with **NMM**; and b) energy profiles Δ*E* for the *endo* Diels-Alder reactions of **Py** (black) and **Me_3N-Py** (red) with **NMM**. Computed at BLYP-D3(BJ)/TZ2P.

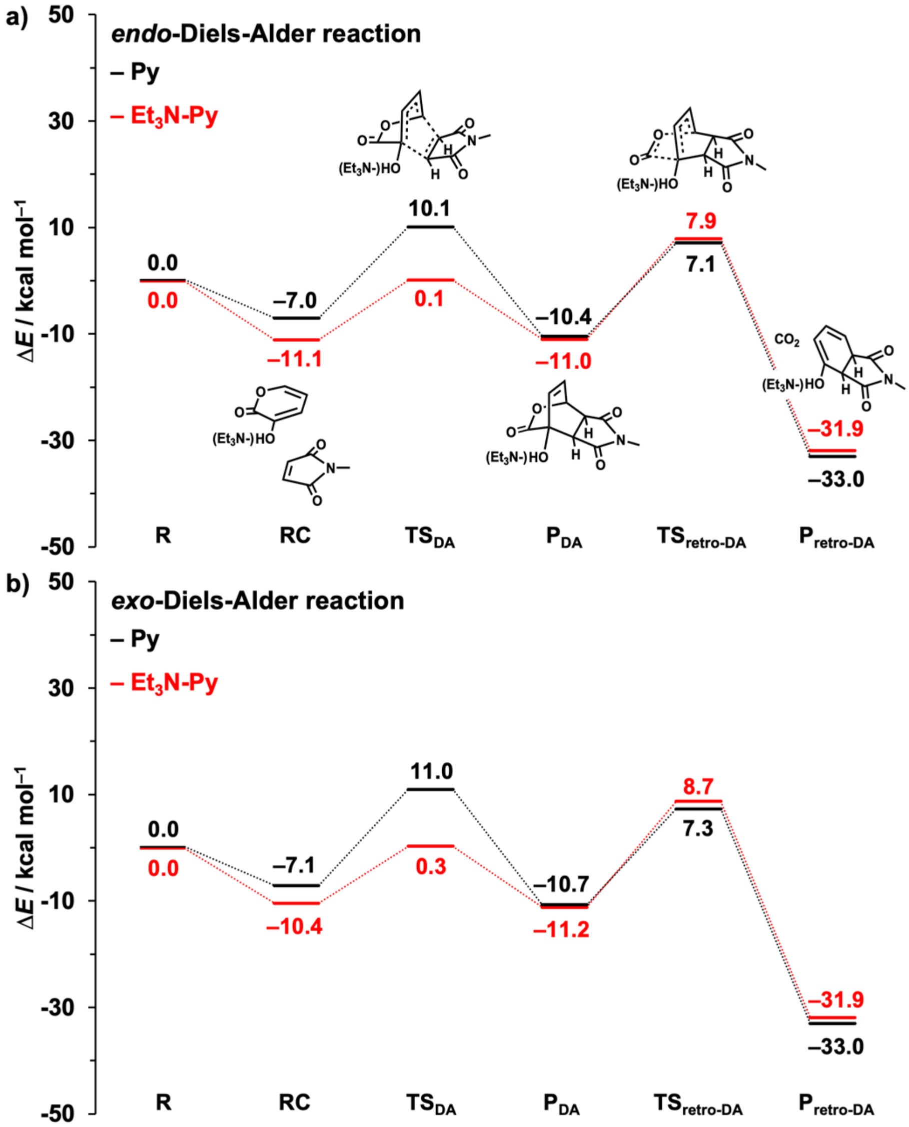

Figure S2. Energy profiles Δ*E* of the a) *endo*- and b) *exo*-Diels-Alder reactions of **Py** (black) and **Et_3N-Py** (red) with **NMM**. Computed at BLYP-D3(BJ)/TZ2P.

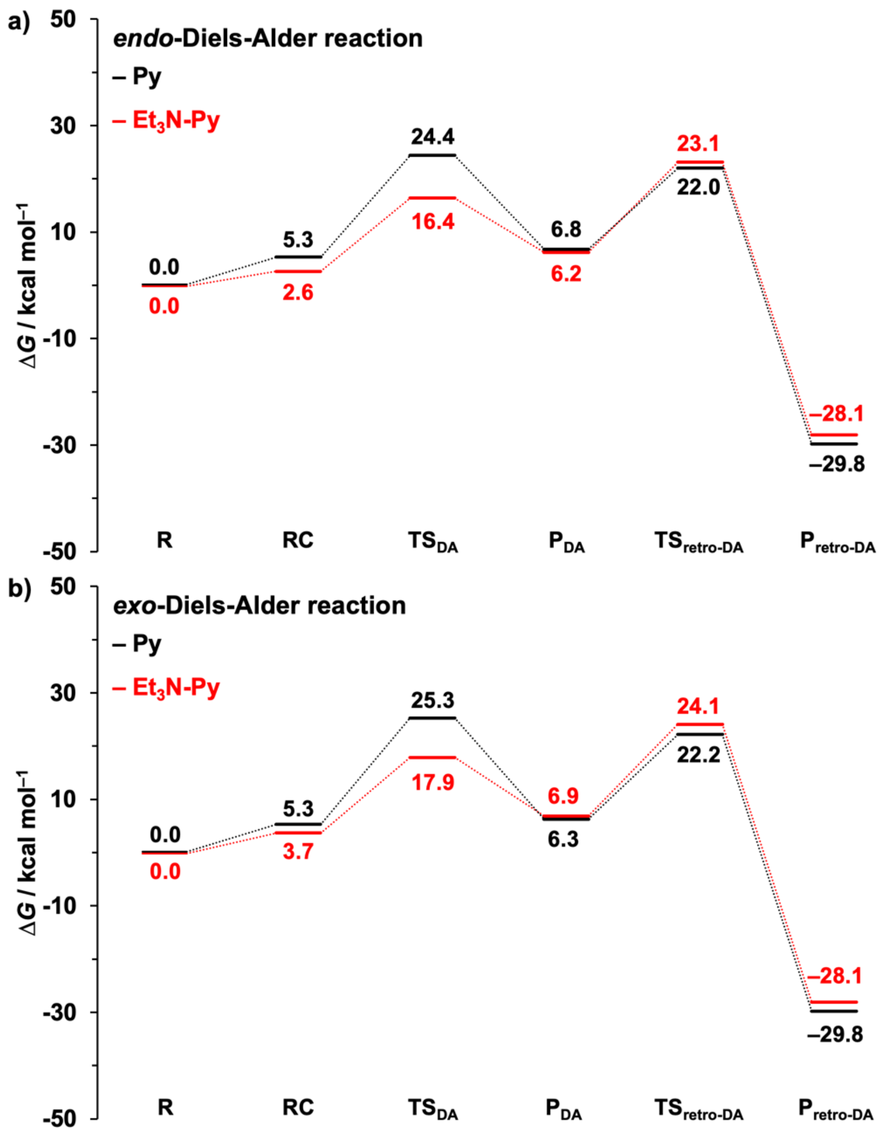

Figure S3. Free energy profiles Δ*G* of the a) *endo*- and b) *exo*-Diels-Alder reactions of **Py** (black) and **Et_3N-Py** (red) with **NMM**. Computed at BLYP-D3(BJ)/TZ2P.

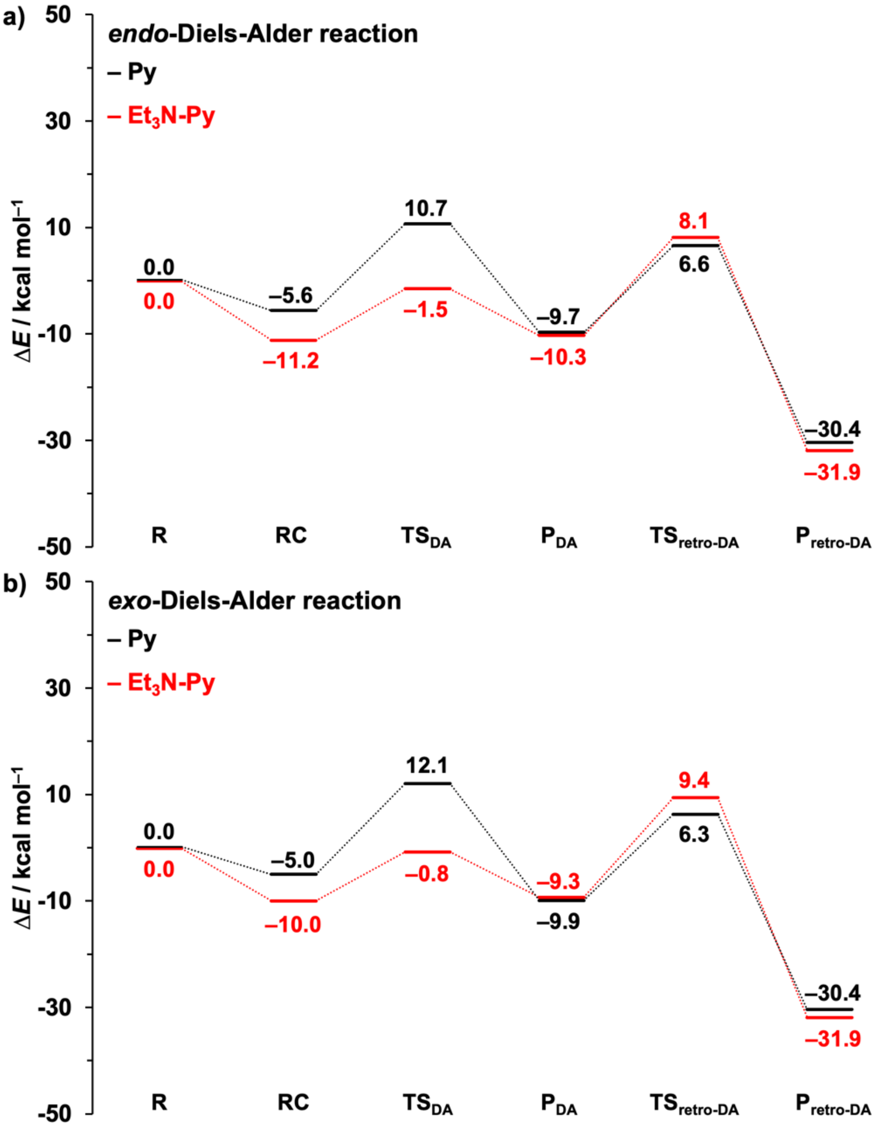

Figure S4. Energy profiles Δ*E* of the a) *endo*-and b) *exo*-Diels-Alder reactions of **Py** (black) and **Et$_3$N-Py** (red) with **NMM**. Computed at COSMO(chloroform)-BLYP-D3(BJ)/TZ2P.

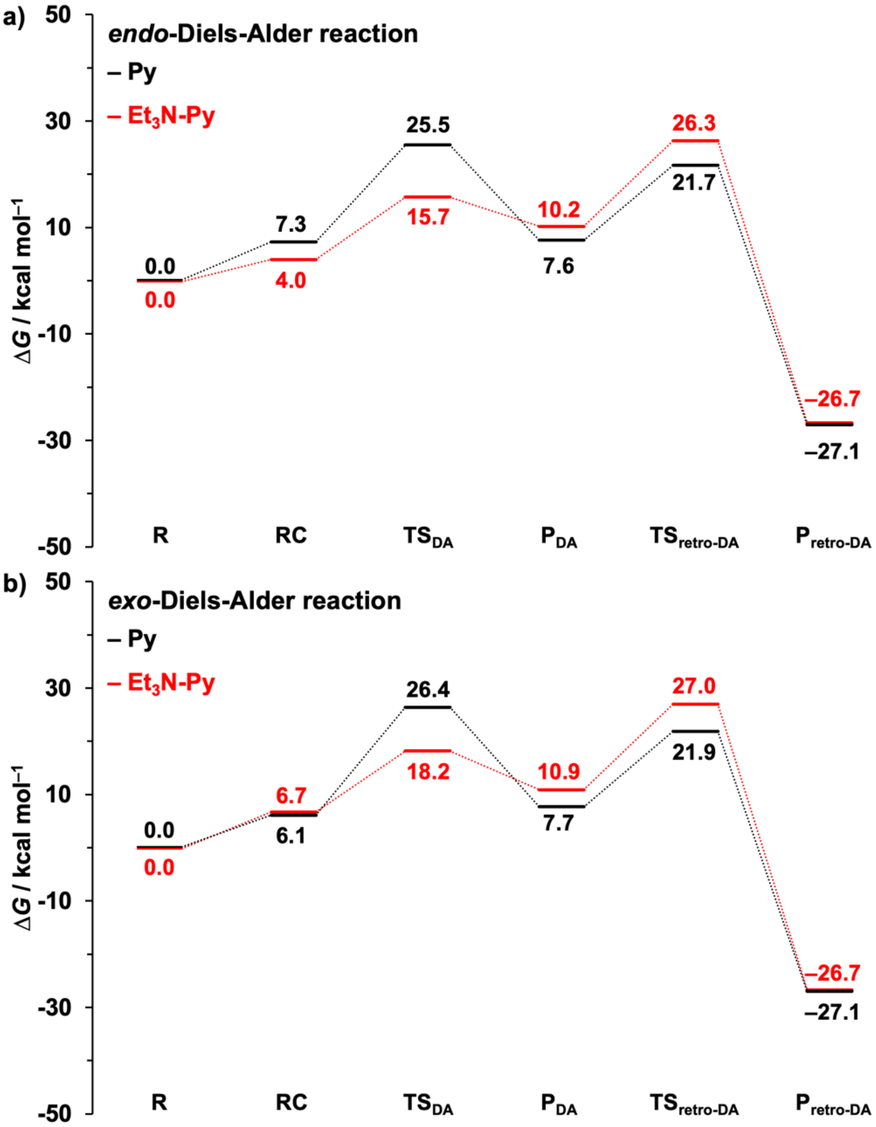

Figure S5. Free energy profiles Δ*G* of the a) *endo*-and b) *exo*-Diels-Alder reactions of **Py** (black) and **Et$_3$N-Py** (red) with **NMM**. Computed at COSMO(chloroform)-BLYP-D3(BJ)/TZ2P.

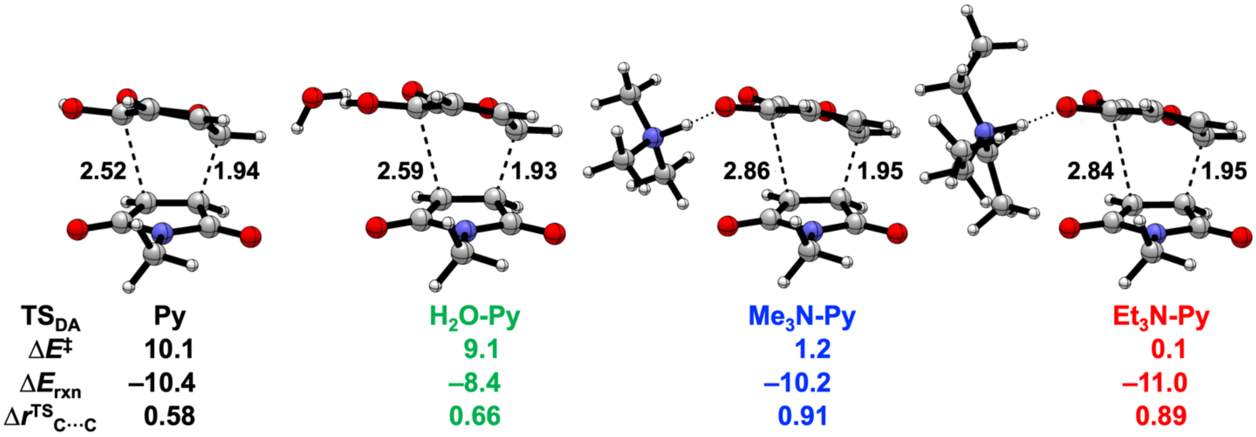

Figure S6. Transition states with the newly forming C···C bond lengths (Å), activation energies ($\Delta E^‡$, kcal mol^{-1}), reaction energies (ΔE_{rxn}, kcal mol^{-1}), and length differences between the two forming bonds ($\Delta r^{TS}_{C\cdots C}$, Å), of the *endo*-Diels-Alder reactions between **(B-)Py** and **NMM**. Computed at BLYP-D3(BJ)/TZ2P.

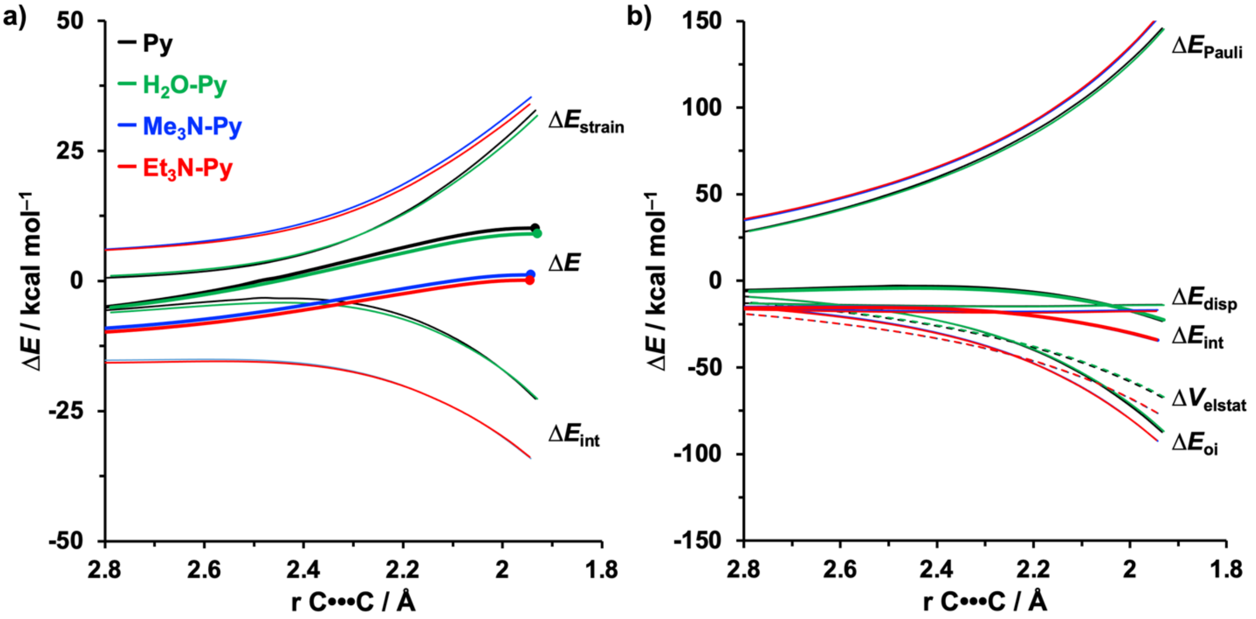

Figure S7. a) Activation strain and b) energy decomposition diagrams of *endo*-Diels-Alder reactions between **(B-)Py** and **NMM** along the IRC, projected on the shorter of the two newly forming C···C bonds (TS indicated by a dot). Computed at BLYP-D3(BJ)/TZ2P.

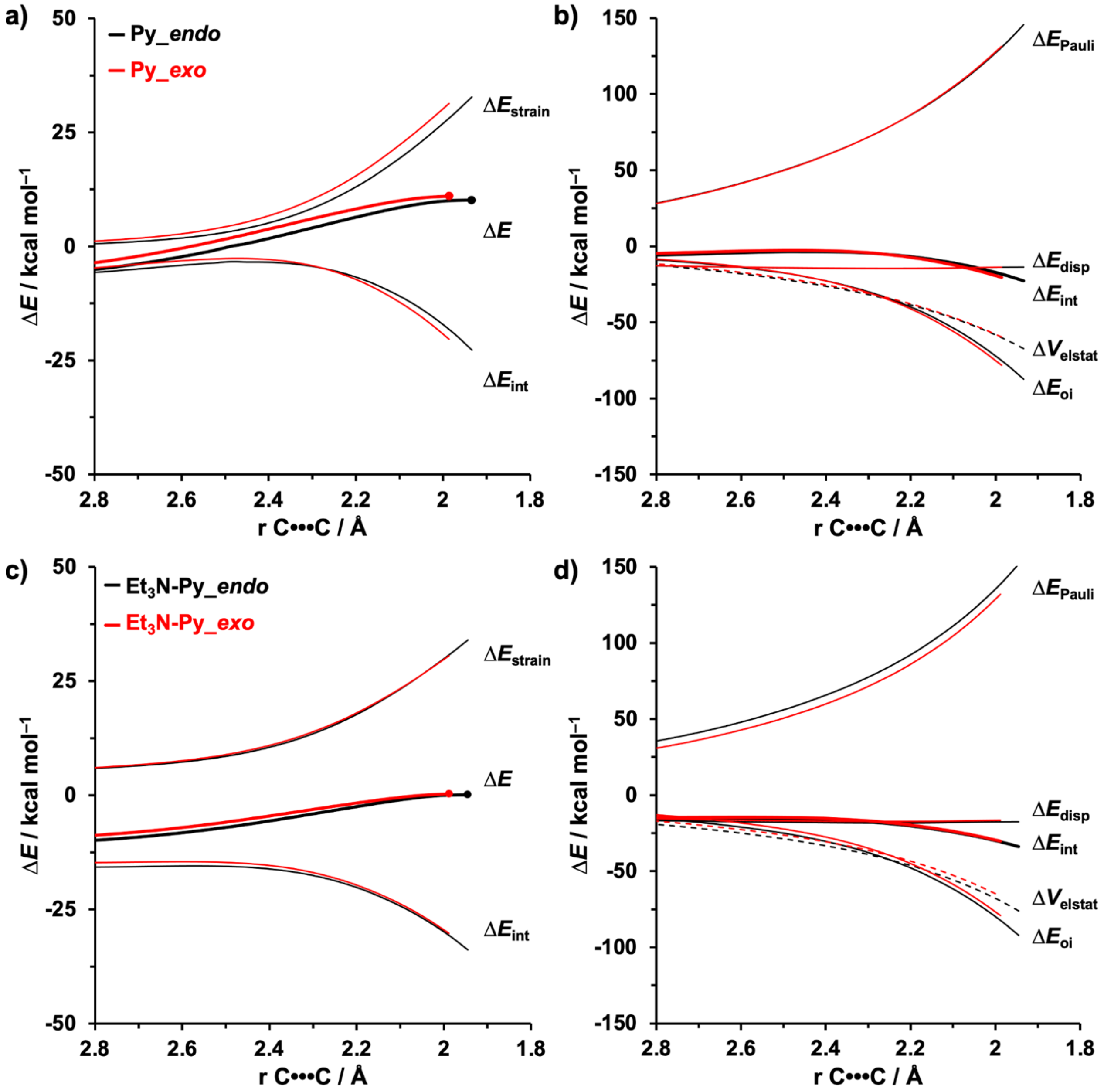

Figure S8. a) Activation strain and b) energy decomposition diagrams of *endo* and *exo*-Diels-Alder reactions between **Py** and **NMM**; c) activation strain and d) energy decomposition diagrams of *endo* and *exo*-Diels-Alder reactions between **Et_3N-Py** and **NMM**. All energy terms are projected on the shorter of the two newly forming C···C bonds (TS indicated by a dot). Computed at BLYP-D3(BJ)/TZ2P.

Table S2. Electronic energies (kcal mol^{-1}), enthalpies (kcal mol^{-1}), Gibbs free energies (kcal mol^{-1}), the number and wavelength of the imaginary vibrational frequencies and Cartesian coordinates (Å), for all stationary points, computed at BLYP-D3(BJ)/TZ2P using ADF2019.

Py (in gas)
E = -1750.92
H = -1694.74
G = -1718.26
N_{imag} = 0

O	0.234614	-2.579372	0.238418
O	1.167240	-0.526265	0.054824
C	-0.206534	1.415811	-0.048175
C	-1.382549	0.613178	0.048932
C	1.012695	0.827210	-0.041746
C	-1.242636	-0.741709	0.146796
C	0.073242	-1.370200	0.151981
H	-0.272155	2.498432	-0.127503
O	-2.277909	-1.605817	0.244704
H	-2.374211	1.063891	0.046183
H	1.969989	1.336279	-0.109512
H	-1.854935	-2.495245	0.298420

Et_3N (in gas)
E = -2614.35
H = -2482.63
G = -2510.81
N_{imag} = 0

C	5.274828	1.084421	-1.620106
C	5.507924	-0.182037	-0.786470
N	4.320956	-1.057476	-0.721999
C	4.688735	-2.455672	-0.423100
C	5.251444	-3.200992	-1.640055
C	3.312627	-0.542960	0.225613
C	1.895583	-1.048435	-0.073884
H	1.822788	-2.139240	0.002050
H	6.191481	1.686110	-1.658328
H	4.986787	0.816454	-2.642758
H	4.483217	1.714667	-1.199288
H	6.321643	-0.753400	-1.244778
H	5.852954	0.090404	0.233044
H	5.409709	-2.502266	0.419914
H	3.786099	-2.975333	-0.085685
H	5.484347	-4.239440	-1.373308
H	4.518588	-3.202701	-2.454626
H	6.173087	-2.741374	-2.014559
H	3.592460	-0.788910	1.271494
H	3.312710	0.549610	0.155349
H	1.181839	-0.618744	0.640035
H	1.597099	-0.758827	-1.087600

Et_3N-Py (in gas)
E = -4377.75
H = -4189.52
G = -4228.18
N_{imag} = 0

O	0.993295	-3.485870	0.191646
O	1.313779	-1.317352	-0.337301
C	-0.449561	0.274396	-0.267321
C	-1.359032	-0.750700	0.127673
C	0.849630	-0.039296	-0.488517
C	-0.929291	-2.047507	0.290968
C	0.482550	-2.385361	0.064996
H	-0.783162	1.299367	-0.395860
O	-1.782467	-3.009985	0.681529
H	-2.406923	-0.522780	0.307376
H	1.634355	0.644241	-0.792443
H	-1.449428	-3.991252	0.609828
H	0.222156	-4.542290	2.440069
H	-0.496676	-5.937618	3.285138
H	0.591160	-5.967611	0.409325
H	-0.267977	-7.317367	1.179523
H	-1.404096	-7.191062	-0.927618
H	-2.682495	-5.990045	-1.125221
H	-0.922210	-5.736099	-2.858476
H	-1.077988	-4.268630	-1.873048
H	0.288830	-5.360128	-1.617850
H	-2.758834	-7.229221	1.252663
H	-2.380738	-5.965909	2.428627
H	-4.722323	-5.768227	1.772526
H	-3.841209	-4.332574	1.202258
C	0.187087	-5.634079	2.485431
C	-0.197230	-6.215099	1.121480
N	-1.472922	-5.673782	0.563679
C	-1.630207	-6.111559	-0.855646
C	-0.777594	-5.321155	-1.853691
C	-2.634305	-6.137522	1.379694
C	-3.952146	-5.413515	1.077233
H	-4.315929	-5.606566	0.062484
H	1.187442	-5.991564	2.755644

Py^- (in gas)
E = -1750.92
H = -1694.74
G = -1718.26
N_{imag} = 0

O	0.338061	-2.588278	0.236579
O	1.175145	-0.523490	0.054484
C	-0.205494	1.411191	-0.047658
C	-1.376574	0.618843	0.048779
C	1.030222	0.843186	-0.043809

C	-1.329039	-0.792828	0.153187
C	0.072232	-1.399667	0.155137
H	-0.272792	2.499182	-0.127288
O	-2.313688	-1.574973	0.242463
H	-2.363168	1.087741	0.044470
H	1.985660	1.357802	-0.112646

Et_3NH^+ (in gas)
E = -2567.12
H = -2425.68
G = -2453.98
N_{imag} = 0

C	5.286745	1.107717	-1.631676
C	5.547843	-0.143326	-0.800176
N	4.318352	-1.057396	-0.727114
C	4.701231	-2.511622	-0.425247
C	5.261568	-3.225533	-1.650412
C	3.264735	-0.525958	0.252644
C	1.867714	-1.046986	-0.066109
H	1.792852	-2.135969	0.011783
H	6.203818	1.704938	-1.662636
H	5.025580	0.852005	-2.665703
H	4.498203	1.739650	-1.211633
H	6.351886	-0.739241	-1.236262
H	5.809910	0.086458	0.236648
H	5.417227	-2.465653	0.400421
H	3.794071	-3.003810	-0.069469
H	5.494299	-4.259761	-1.376853
H	4.526775	-3.257755	-2.463962
H	6.184135	-2.769437	-2.022438
H	3.608184	-0.821518	1.248226
H	3.298611	0.563051	0.184179
H	1.161760	-0.617634	0.652162
H	1.546058	-0.738484	-1.068195
H	3.886373	-1.050885	-1.661568

Py (in chloroform)
E = -1756.58
H = -1700.78
G = -1724.41
N_{imag} = 0

O	0.270415	-2.587148	0.237216
O	1.173687	-0.520876	0.054638
C	-0.204225	1.421267	-0.048826
C	-1.373684	0.607192	0.049260
C	1.020202	0.841685	-0.043151
C	-1.232031	-0.749012	0.147196
C	0.080995	-1.372597	0.152407
H	-0.281727	2.500486	-0.127733
O	-2.295992	-1.600037	0.244820
H	-2.364218	1.054043	0.046779

H	1.974960	1.348053	-0.110556
H	-1.921532	-2.506865	0.301274

Et_3N (in chloroform)
E = -2614.67
H = -2483.17
G = -2511.33
N_{imag} = 0

C	5.279834	1.091972	-1.615607
C	5.505529	-0.184777	-0.796226
N	4.312575	-1.057435	-0.740279
C	4.681797	-2.455826	-0.430331
C	5.258789	-3.207587	-1.636294
C	3.309989	-0.540027	0.216230
C	1.890891	-1.049349	-0.065228
H	1.822865	-2.140692	0.007611
H	6.198488	1.691315	-1.638817
H	5.002102	0.838781	-2.645362
H	4.486004	1.718063	-1.192756
H	6.317373	-0.755029	-1.258592
H	5.847289	0.075140	0.226833
H	5.395966	-2.491980	0.417985
H	3.778218	-2.975910	-0.096995
H	5.498375	-4.240902	-1.356029
H	4.530598	-3.227968	-2.455436
H	6.178645	-2.743428	-2.009632
H	3.602353	-0.784322	1.258258
H	3.307846	0.552069	0.142949
H	1.187379	-0.623603	0.661058
H	1.574000	-0.754648	-1.072385

Et_3N-Py (in chloroform)
E = -4381.54
H = -4193.36
G = -4234.17
N_{imag} = 0

O	1.134863	-3.425175	0.471227
O	1.294419	-1.361242	-0.411975
C	-0.547859	0.135049	-0.501153
C	-1.381021	-0.864711	0.080617
C	0.757401	-0.141840	-0.733762
C	-0.875952	-2.099732	0.420337
C	0.542944	-2.389167	0.192408
H	-0.944554	1.109912	-0.765846
O	-1.650091	-3.028871	1.012126
H	-2.432835	-0.662627	0.267988
H	1.492267	0.523093	-1.171701
H	-1.410939	-4.044370	0.815891
H	0.222472	-4.706343	2.587448
H	-0.539568	-6.156274	3.290684
H	0.595994	-5.952357	0.439236

H	-0.303650	-7.343749	1.072566
H	-1.416299	-7.004500	-1.025621
H	-2.652559	-5.749976	-1.137848
H	-0.855515	-5.405102	-2.813190
H	-0.979444	-4.024234	-1.709772
H	0.346396	-5.185772	-1.529378
H	-2.781779	-7.216190	1.121446
H	-2.412845	-6.067750	2.412131
H	-4.735712	-5.775491	1.739644
H	-3.828299	-4.312402	1.296400
C	0.165413	-5.797269	2.533898
C	-0.207258	-6.244638	1.117400
N	-1.464943	-5.625916	0.593586
C	-1.610391	-5.930256	-0.864430
C	-0.714242	-5.084678	-1.774378
C	-2.649583	-6.143744	1.348444
C	-3.951716	-5.378708	1.084093
H	-4.302070	-5.486524	0.052352
H	1.152500	-6.202167	2.785930

Py^- (in chloroform)
E = -1743.58
H = -1696.18
G = -1719.76
N_{imag} = 0

O	0.327839	-2.591417	0.237183
O	1.168647	-0.525536	0.054870
C	-0.203767	1.416455	-0.047992
C	-1.374812	0.614806	0.048968
C	1.030084	0.849872	-0.044296
C	-1.314626	-0.787304	0.152270
C	0.069171	-1.388988	0.154440
H	-0.278385	2.499288	-0.127105
O	-2.313626	-1.585669	0.243224
H	-2.357112	1.085163	0.044500
H	1.987153	1.352039	-0.112367

Et_3NH^+ (in chloroform)
E = -2606.1
H = -2464.78
G = -2493.88
N_{imag} = 0

C	5.283059	1.106347	-1.623332
C	5.546668	-0.152174	-0.805697
N	4.314943	-1.057319	-0.734950
C	4.691456	-2.508077	-0.426842
C	5.265375	-3.219236	-1.646159
C	3.268994	-0.520274	0.244323
C	1.874549	-1.052173	-0.062976
H	1.808297	-2.139576	0.036134
H	6.205230	1.694340	-1.662704

H	5.000838	0.855606	-2.652391
H	4.504405	1.737286	-1.184773
H	6.343743	-0.746464	-1.254145
H	5.815140	0.066097	0.230757
H	5.397081	-2.461827	0.406176
H	3.780064	-2.999652	-0.084338
H	5.483977	-4.256065	-1.372531
H	4.542009	-3.234977	-2.469575
H	6.195822	-2.764701	-1.998869
H	3.619528	-0.804619	1.239415
H	3.296365	0.567047	0.163103
H	1.169627	-0.609380	0.647439
H	1.557512	-0.766410	-1.072586
H	3.883250	-1.050945	-1.668870

uncatalyzed_RC_*endo* (in gas)
E = -3634.75
H = -3514.64
G = -3551.71
N_{imag} = 0

O	2.629308	0.108138	1.583187
N	0.313930	0.020027	1.924819
O	-1.913379	-0.676542	1.791837
C	1.537840	-0.397675	1.370160
C	1.204883	-1.567560	0.482930
C	-0.115993	-1.792149	0.532765
C	-0.743264	-0.791025	1.466728
C	0.154043	1.138536	2.846310
H	1.968051	-2.093443	-0.077848
H	-0.702917	-2.547061	0.023203
H	-0.306343	0.799047	3.779329
H	-0.482511	1.911860	2.402864
H	1.147949	1.544718	3.046527
O	-1.237908	-1.653715	-3.069484
O	0.695561	-0.557578	-2.614590
C	0.689285	1.373451	-1.209835
C	-0.728862	1.297397	-1.080322
C	1.349128	0.452146	-1.955029
C	-1.395898	0.280822	-1.708502
C	-0.686206	-0.717276	-2.507573
H	1.256789	2.147716	-0.704857
O	-2.740555	0.122382	-1.641763
H	-1.279905	2.020184	-0.485163
H	2.419319	0.412567	-2.117769
H	-2.944398	-0.684934	-2.165826

uncatalyzed_TS$_{DA}$_*endo* (in gas)
E = -3617.57
H = -3497.59
G = -3532.62
N_{imag} = 1, ν = i460.111877 cm^{-1}

O	2.601507	0.247592	1.506296
N	0.289412	0.124788	1.835764
O	-1.924880	-0.635929	1.781404
C	1.496540	-0.176901	1.199936
C	1.135671	-1.084133	0.037224
C	-0.243324	-1.390721	0.167347
C	-0.796602	-0.640886	1.310861
C	0.157475	1.025847	2.975076
H	1.866488	-1.830244	-0.262521
H	-0.751976	-2.258259	-0.231654
H	-0.336048	0.506155	3.801831
H	-0.445241	1.899663	2.704400
H	1.161057	1.340933	3.268373
O	-1.261473	-1.679985	-3.102036
O	0.721587	-0.759274	-2.491731
C	0.707612	1.273727	-1.159956
C	-0.666994	1.282876	-1.135906
C	1.352112	0.051118	-1.518856
C	-1.325063	0.106286	-1.546891
C	-0.649842	-0.861815	-2.430313
H	1.302224	2.088110	-0.760078
O	-2.663088	0.002165	-1.483514
H	-1.249004	2.117163	-0.754982
H	2.423768	0.040868	-1.689898
H	-2.903395	-0.789552	-2.020560

uncatalyzed_Product$_{DA}$_*endo* (in gas)
E = -3638.15
H = -3516.35
G = -3550.31
N_{imag} = 0

O	2.531172	0.171771	1.556874
N	0.230169	0.207385	1.917930
O	-2.067498	-0.052000	1.750533
C	1.394852	-0.118483	1.221158
C	0.982320	-0.881386	-0.041330
C	-0.561160	-0.950022	-0.002163
C	-0.955871	-0.230325	1.295131
C	0.222535	0.956007	3.175247
H	1.458853	-1.866401	-0.041882
H	-0.954175	-1.970230	0.020327
H	-0.332302	0.395133	3.932520
H	-0.264957	1.925202	3.029762
H	1.259992	1.097833	3.482962
O	-1.089760	-1.640279	-3.297382
O	0.892732	-0.937469	-2.459198
C	0.746579	1.240464	-1.351143
C	-0.588956	1.174807	-1.342275
C	1.410974	-0.114979	-1.328116
C	-1.139502	-0.239920	-1.306237
C	-0.463311	-1.011490	-2.469683

H	1.333057	2.153303	-1.354678
O	-2.536898	-0.313081	-1.407199
H	-1.280125	2.011617	-1.356847
H	2.490851	-0.097126	-1.460854
H	-2.721077	-0.912290	-2.162452

uncatalyzed_$TS_{retro\text{-}DA}$_*endo* (in gas)
E = -3620.62
H = -3500.57
G = -3535.09
N_{imag} = 1, ν = i427.413879 cm^{-1}

O	2.532130	0.178344	1.767988
N	0.223586	0.160487	2.008988
O	-2.059141	-0.179057	1.741488
C	1.418755	-0.065905	1.338836
C	1.071438	-0.665876	-0.049254
C	-0.466305	-0.870427	-0.030160
C	-0.928489	-0.266880	1.312274
C	0.171893	0.744464	3.351433
H	1.631341	-1.597412	-0.164860
H	-0.746039	-1.928041	-0.032725
H	-0.879586	0.825667	3.632334
H	0.645055	1.730573	3.342166
H	0.706905	0.103142	4.058317
O	-1.249421	-1.761108	-3.194953
O	0.858968	-1.034141	-2.645568
C	0.668576	1.405712	-1.438270
C	-0.691286	1.157430	-1.489373
C	1.491149	0.319978	-1.107108
C	-1.147137	-0.189868	-1.251000
C	-0.386154	-1.127714	-2.595379
H	1.093326	2.313785	-1.857060
O	-2.511145	-0.408161	-1.362521
H	-1.399175	1.879944	-1.888687
H	2.548700	0.343945	-1.354046
H	-2.596562	-1.071533	-2.094256

uncatalyzed_$Product_{retro\text{-}DA}$_*endo* (in gas)
E = -3147.6
H = -3036.18
G = -3067.71
N_{imag} = 0

O	2.439771	0.309283	1.815859
N	0.136306	0.079408	2.048140
O	-2.119419	-0.340538	1.690010
C	1.362524	0.003463	1.346749
C	1.068515	-0.548320	-0.067369
C	-0.479619	-0.698012	-0.110705
C	-0.954385	-0.323351	1.300311
C	0.027839	0.544139	3.433162
H	1.544498	-1.539350	-0.089294

H	-0.790108	-1.737429	-0.289433
H	-0.419396	-0.238119	4.053486
H	-0.605242	1.435388	3.476473
H	1.036158	0.777050	3.779885
H	-1.119935	1.543141	-2.714107
H	2.759417	0.377506	-1.173712
C	0.905070	0.990598	-2.021210
C	-0.552087	0.948889	-2.003131
C	1.675836	0.315909	-1.148901
C	-1.211498	0.180089	-1.114509
H	-2.845238	-0.191337	-0.177746
H	1.379418	1.608902	-2.781049
O	-2.577817	0.106386	-1.078045

uncatalyzed_RC_*exo* (in gas)
E = -3634.83
H = -3514.73
G = -3551.73
N_{imag} = 0

O	-1.968872	-0.284427	-1.133020
N	0.237503	-0.786137	-0.538516
O	2.528517	-0.518859	-0.122776
C	-0.799673	0.041581	-1.002625
C	-0.166380	1.380645	-1.284939
C	1.139627	1.308676	-0.997363
C	1.452728	-0.078970	-0.497749
C	0.055446	-2.163535	-0.083067
H	-0.746240	2.214970	-1.660954
H	1.906660	2.069314	-1.079232
H	-0.438539	-2.748582	-0.864228
H	1.046280	-2.572827	0.124465
H	-0.554422	-2.178309	0.825132
O	-1.634828	-0.462582	2.277689
O	0.347849	0.619870	2.464845
C	0.499422	2.961174	2.041447
C	-0.890736	3.009158	1.720982
C	1.072156	1.781210	2.384022
C	-1.624159	1.854875	1.769640
C	-1.019585	0.589868	2.182575
H	1.112535	3.855464	1.999069
O	-2.943329	1.801193	1.460479
H	-1.366460	3.941219	1.427527
H	2.115889	1.617294	2.623365
H	-3.202911	0.854246	1.519676

uncatalyzed_TS_{DA}_*exo* (in gas)
E = -3616.67
H = -3496.78
G = -3531.75
N_{imag} = 0, ν = i490.39115 cm^{-1}

O	-1.936172	-0.485190	-1.044972

N	0.298544	-0.881559	-0.448964
O	2.559671	-0.548019	0.021276
C	-0.832622	-0.063468	-0.729270
C	-0.382474	1.333826	-0.555034
C	0.982553	1.339321	-0.179635
C	1.435753	-0.118187	-0.186794
C	0.250641	-2.338387	-0.366770
H	-0.935234	2.145818	-1.006858
H	1.695109	2.078798	-0.530932
H	-0.667835	-2.667310	-0.857235
H	1.126872	-2.758519	-0.867317
H	0.242159	-2.661675	0.679891
O	-1.807318	-0.240900	2.296017
O	0.271533	0.667523	2.267549
C	0.510853	3.037793	1.830633
C	-0.849217	3.171417	1.668304
C	1.056477	1.723453	1.763640
C	-1.596758	1.984688	1.502505
C	-1.088616	0.711895	2.040365
H	1.180570	3.892333	1.838614
O	-2.914262	2.021093	1.231999
H	-1.344460	4.134612	1.581883
H	2.098083	1.531642	2.001698
H	-3.246638	1.098916	1.341146

uncatalyzed_Product$_{DA}$_*exo* (in gas)
E = -3638.39
H = -3516.68
G = -3550.75
N_{imag} = 0

O	-2.082234	-0.713028	0.394846
N	0.088557	-0.913847	-0.407737
O	2.281846	-0.495110	-1.076121
C	-1.017694	-0.207573	0.073412
C	-0.638225	1.270684	0.169732
C	0.831619	1.338602	-0.290363
C	1.209949	-0.103930	-0.655313
C	0.092477	-2.370371	-0.563003
H	-1.328173	1.871067	-0.428473
H	0.990468	1.979634	-1.162258
H	-0.758456	-2.675085	-1.178091
H	1.033528	-2.649126	-1.039979
H	0.010657	-2.847530	0.418510
O	-0.136287	0.109618	3.369744
O	1.489146	0.916854	2.018411
C	1.129662	3.224384	1.300274
C	-0.142694	3.173961	1.707389
C	1.680031	1.876152	0.899900
C	-0.755738	1.789104	1.672005
C	0.194091	0.855904	2.481591

H	1.736367	4.122474	1.246278
O	-2.073483	1.805161	2.149259
H	-0.745047	4.006229	2.058333
H	2.748813	1.849984	0.691802
H	-2.416188	0.889307	2.128995

uncatalyzed_TS$_{retro\text{-}DA}$_*exo* (in gas)
E = -3620.39
H = -3500.54
G = -3534.89
N_{imag} = 0, ν = i413.10859 cm^{-1}

O	-2.084590	-0.809138	0.292790
N	0.123218	-0.871009	-0.436364
O	2.273606	-0.315383	-1.124406
C	-1.042876	-0.238595	0.039999
C	-0.745589	1.264996	0.162104
C	0.674001	1.430765	-0.424649
C	1.174824	-0.000491	-0.707826
C	0.241070	-2.323036	-0.583377
H	-1.509714	1.813711	-0.396712
H	0.658554	1.949056	-1.395420
H	-0.765882	-2.742847	-0.555757
H	0.734989	-2.550179	-1.531224
H	0.836853	-2.733089	0.238750
O	-0.336618	0.019698	3.318532
O	1.450949	0.672327	2.031256
C	1.087310	3.264790	1.238855
C	-0.165219	3.074753	1.797592
C	1.591988	2.211976	0.471529
C	-0.809889	1.793600	1.639031
C	0.283319	0.671463	2.484029
H	1.734616	4.072531	1.569589
O	-2.057626	1.682298	2.234192
H	-0.586005	3.766643	2.523800
H	2.657585	2.114338	0.278841
H	-1.995783	0.884197	2.814168

uncatalyzed_Product$_{retro\text{-}DA}$_*exo* (in gas)
E = -3147.6
H = -3036.18
G = -3067.71
N_{imag} = 0

O	-2.107610	-0.608286	0.428737
N	0.099122	-0.828646	-0.260102
O	2.283872	-0.427704	-0.951713
C	-1.037834	-0.117784	0.075920
C	-0.731154	1.383726	-0.014828
C	0.729365	1.464747	-0.545376
C	1.192892	-0.008267	-0.623225
C	0.162938	-2.291945	-0.236136
H	-1.452984	1.817755	-0.721435

H	0.733792	1.818461	-1.586545
H	-0.585838	-2.708026	-0.916633
H	1.167243	-2.580556	-0.551144
H	-0.038350	-2.654690	0.776176
H	-2.646403	1.050030	1.426611
H	-0.273715	3.154510	2.962711
C	1.296901	2.869739	1.433591
C	-0.036341	2.708001	2.000759
C	1.678483	2.313831	0.269287
C	-0.986458	2.010758	1.347523
H	2.683381	2.436247	-0.122824
H	2.011531	3.468153	1.995630
O	-2.252128	1.850860	1.843626

catalyzed_RC_*endo* (in gas)
E = -6265.63
H = -6011.38
G = -6064.39
N_{imag} = 0

O	2.216507	0.971371	2.483249
N	0.091646	0.162867	1.920076
O	-1.603633	-1.148853	0.986103
C	1.500437	0.175286	1.889727
C	1.894217	-0.967559	1.001169
C	0.778894	-1.575354	0.551074
C	-0.413018	-0.881112	1.122552
C	-0.735533	1.151428	2.596147
H	2.931106	-1.217366	0.817994
H	0.692668	-2.433175	-0.102925
H	-0.065482	1.836839	3.120092
H	-1.405408	0.665160	3.312910
H	-1.338537	1.703263	1.866711
O	-0.154927	-1.477832	-2.966138
O	1.720086	-0.453030	-2.260198
C	1.649943	1.560746	-0.989113
C	0.238250	1.569679	-1.044786
C	2.351104	0.551302	-1.577943
C	-0.473549	0.549118	-1.683796
C	0.315710	-0.535199	-2.342919
H	2.195068	2.337870	-0.461720
O	-1.761452	0.491529	-1.749843
H	-0.328480	2.369653	-0.574694
H	3.429382	0.450994	-1.605713
H	-2.533320	-0.753898	-2.027674
H	-2.319416	-0.173166	-4.391612
H	-3.088974	-1.597187	-5.127505
H	-4.425400	0.200737	-3.017606
H	-5.038591	-1.376856	-3.555867
H	-5.076390	-2.132450	-1.138280
H	-3.585122	-1.896659	-0.189196
H	-5.094847	-0.182594	0.522351

H	-5.458327	0.353330	-1.122859
H	-3.773702	-3.377748	-2.968055
H	-2.199154	-2.668399	-3.418268
H	-1.682536	-4.430427	-1.901219
H	-1.456059	-2.928010	-0.989207
C	-3.278339	-0.640788	-4.632290
C	-4.130700	-0.788077	-3.370766
N	-3.392819	-1.438100	-2.221225
C	-4.225887	-1.462544	-0.955447
C	-4.680124	-0.079663	-0.486348
C	-2.902348	-2.818758	-2.601385
C	-2.199094	-3.566208	-1.470052
H	-2.892147	-3.931809	-0.706817
H	-3.812620	0.002739	-5.340049
H	-3.825945	0.601913	-0.439697

catalyzed_TS_{DA}_*endo* (in gas)
E = -6254.44
H = -6000.1
G = -6050.57
N_{imag} = 0, ν = i428.76412 cm^{-1}

O	2.481992	0.655396	2.256673
N	0.241596	0.107788	1.875707
O	-1.704132	-0.953352	1.085809
C	1.611360	0.074294	1.616465
C	1.775315	-0.784502	0.371345
C	0.493680	-1.326269	0.092154
C	-0.483587	-0.766534	0.994845
C	-0.386320	0.919071	2.906419
H	2.686055	-1.373155	0.298609
H	0.280472	-2.152599	-0.568199
H	0.406605	1.333929	3.533168
H	-1.061213	0.301820	3.507460
H	-0.967285	1.734602	2.458253
O	-0.082524	-1.171782	-3.207667
O	1.718989	-0.267355	-2.194931
C	1.487904	1.646810	-0.737566
C	0.148386	1.725237	-1.004443
C	2.189408	0.448572	-1.079701
C	-0.516121	0.650541	-1.678887
C	0.341321	-0.366492	-2.387812
H	2.001359	2.393464	-0.138954
O	-1.778899	0.594138	-1.796334
H	-0.462541	2.557914	-0.664253
H	3.275465	0.461994	-1.095642
H	-2.562452	-0.743106	-2.035886
H	-2.207096	-0.454742	-4.477579
H	-2.991308	-1.929757	-5.075597
H	-4.359832	0.101750	-3.211725
H	-4.968851	-1.523136	-3.590933
H	-5.064639	-2.125100	-1.134876

H	-3.632831	-1.707325	-0.148503
H	-5.277129	-0.025119	0.306200
H	-5.564925	0.329855	-1.402143
H	-3.553597	-3.409423	-2.980625
H	-1.937855	-2.666797	-3.123891
H	-1.696077	-4.403695	-1.499098
H	-1.731433	-2.906266	-0.545444
C	-3.172552	-0.921819	-4.691680
C	-4.063057	-0.920709	-3.448665
N	-3.367927	-1.448215	-2.209006
C	-4.261247	-1.394013	-0.982068
C	-4.807730	0.001937	-0.683089
C	-2.783998	-2.830488	-2.455874
C	-2.321165	-3.557522	-1.193922
H	-3.157328	-3.951147	-0.608111
H	-3.676140	-0.348918	-5.478099
H	-3.992397	0.730561	-0.651938

catalyzed_Product$_{DA}$_*endo* (in gas)

E = -6265.5

H = -6010.34

G = -6060.81

N_{imag} = 0

O	2.337616	0.441462	2.700985
N	0.089900	0.748347	2.204202
O	-2.037153	0.647605	1.272286
C	1.366902	0.229070	1.992042
C	1.319594	-0.621842	0.720743
C	-0.131349	-0.510876	0.205124
C	-0.858002	0.349444	1.238132
C	-0.234540	1.620221	3.332714
H	1.616947	-1.648320	0.956817
H	-0.640630	-1.474270	0.126455
H	-1.319914	1.731859	3.362588
H	0.238765	2.598862	3.200639
H	0.136131	1.172646	4.258858
O	0.455468	-1.455108	-3.049585
O	2.061707	-0.918282	-1.559676
C	1.878169	1.360089	-0.697346
C	0.629093	1.463311	-1.161140
C	2.276522	-0.058888	-0.372343
C	-0.126782	0.151087	-1.262865
C	0.792752	-0.819272	-2.078375
H	2.561125	2.187773	-0.536754
O	-1.379043	0.333067	-1.818077
H	0.123873	2.376032	-1.461801
H	3.324825	-0.186571	-0.107491
H	-1.856985	-0.552974	-1.986839
N	-2.992731	-1.803855	-2.384799
C	-3.985722	-1.916548	-1.278139
C	-3.632760	-1.221148	-3.601249

C	-2.400604	-3.132035	-2.703494
C	-4.564519	-0.579266	-0.800962
H	-3.474901	-2.387138	-0.433666
H	-4.801442	-2.600480	-1.583797
C	-2.665774	-0.959331	-4.762573
H	-4.077006	-0.269287	-3.301034
H	-4.460345	-1.877935	-3.932345
H	-1.718763	-2.986300	-3.542451
H	-3.201478	-3.821044	-3.035409
C	-1.609140	-3.777834	-1.561949
H	-1.144196	-4.699537	-1.929894
H	-2.232343	-4.043635	-0.702450
H	-0.802466	-3.118955	-1.227085
H	-1.787852	-0.404084	-4.419723
H	-3.181353	-0.362983	-5.524506
H	-2.323863	-1.881087	-5.242983
H	-3.766720	0.122594	-0.542382
H	-5.162190	-0.755809	0.101081
H	-5.223355	-0.118711	-1.543809

catalyzed_$TS_{retro-DA}$_*endo* (in gas)
E = -6246.64
H = -5993.1
G = -6043.85
N_{imag} = 0, ν = i525.59531 cm^{-1}

O	2.124985	0.277077	2.918350
N	-0.043604	0.707434	2.213424
O	-2.081043	0.698661	1.103476
C	1.239087	0.182227	2.086897
C	1.323986	-0.514744	0.709336
C	-0.102927	-0.419859	0.112802
C	-0.905997	0.389742	1.145766
C	-0.475575	1.460061	3.392415
H	1.657203	-1.544351	0.864073
H	-0.579881	-1.398774	0.015433
H	-1.507202	1.772871	3.221861
H	0.173047	2.329960	3.529896
H	-0.412423	0.829490	4.284658
O	0.376238	-1.429276	-3.158434
O	2.098955	-1.084346	-1.693048
C	1.962554	1.431394	-0.769044
C	0.709722	1.457254	-1.346975
C	2.329961	0.227278	-0.139328
C	-0.105370	0.264237	-1.291730
C	0.962511	-0.921499	-2.218837
H	2.709132	2.186885	-0.996352
O	-1.314648	0.368140	-1.911167
H	0.386757	2.265840	-1.998772
H	3.375761	0.020919	0.067549
H	-1.803108	-0.530184	-1.999325
N	-2.942665	-1.777272	-2.318198

C	-3.876954	-1.901245	-1.162077
C	-3.651694	-1.213738	-3.506209
C	-2.348200	-3.100568	-2.657655
C	-4.551591	-0.591054	-0.740270
H	-3.293044	-2.275623	-0.316277
H	-4.643535	-2.667203	-1.389913
C	-2.756442	-0.989141	-4.731031
H	-4.066076	-0.250499	-3.200024
H	-4.504707	-1.868215	-3.770217
H	-1.692175	-2.947282	-3.514897
H	-3.155431	-3.793243	-2.965275
C	-1.519883	-3.744414	-1.542043
H	-1.067150	-4.665732	-1.925811
H	-2.114626	-4.010444	-0.662619
H	-0.701020	-3.086314	-1.236295
H	-1.843952	-0.453815	-4.453186
H	-3.307022	-0.388346	-5.464528
H	-2.470827	-1.924992	-5.220895
H	-3.812243	0.201951	-0.600466
H	-5.056134	-0.747808	0.220221
H	-5.308399	-0.261329	-1.458668

catalyzed_Product$_{retro\text{-}DA}$_*endo* (in gas)
E = -5773.33
H = -5529.53
G = -5575.93
N_{imag} = 0

O	-1.161598	-1.660619	0.254594
N	1.002656	-1.066914	-0.310312
O	2.955269	0.087095	-0.829181
C	-0.344775	-0.805368	-0.040978
C	-0.560133	0.722225	-0.217192
C	0.881080	1.304612	-0.241224
C	1.775750	0.085110	-0.522817
C	1.549140	-2.422792	-0.344772
H	-1.045814	0.841951	-1.194961
H	1.032917	2.026417	-1.055455
H	0.997245	-3.028556	-1.069801
H	2.599603	-2.346838	-0.631756
H	1.457527	-2.887673	0.642135
H	-1.606988	-2.061316	3.780557
H	-1.464134	-0.424062	3.121872
C	0.416311	2.164887	2.058998
C	-1.011126	1.909469	1.925077
C	1.309574	1.912686	1.082211
C	-1.499719	1.261800	0.838227
H	-2.192541	-0.687249	4.723564
H	0.763479	2.615583	2.987868
O	-2.792663	0.969136	0.594109
H	-1.689373	2.268713	2.694393
H	2.365154	2.143955	1.193347

H	-3.301963	0.696281	1.462026
N	-4.164031	-0.026660	2.633740
C	-4.366426	0.883604	3.792990
C	-3.462748	-1.287272	3.027094
C	-5.466574	-0.343754	1.974655
H	-3.396719	1.001904	4.281859
H	-5.045495	0.412622	4.528196
C	-4.887228	2.272933	3.402745
C	-2.105310	-1.088236	3.708355
H	-3.297667	-1.851748	2.107407
H	-4.128545	-1.884384	3.677523
H	-6.129163	-0.847852	2.702685
C	-5.339961	-1.188079	0.699897
H	-5.935522	0.609277	1.716236
H	-4.599829	-0.759044	0.017483
H	-6.311017	-1.209558	0.191828
H	-5.055224	-2.223116	0.910447
H	-4.843976	2.933867	4.275995
H	-5.925744	2.252880	3.058586
H	-4.271260	2.712320	2.610082

catalyzed_RC_*exo* (in gas)
E = -6264.91
H = -6010.51
G = -6063.3
N_{imag} = 0

O	-1.786255	-0.773273	-0.325352
N	0.549133	-0.850010	-0.202831
O	2.800992	-0.262344	-0.466529
C	-0.687579	-0.260451	-0.513470
C	-0.369860	1.066696	-1.130549
C	0.966924	1.211804	-1.183212
C	1.611587	-0.005345	-0.591391
C	0.719347	-2.107777	0.514133
H	-1.149029	1.740277	-1.462731
H	1.552857	2.032148	-1.577202
H	0.625649	-1.946529	1.593919
H	-0.047507	-2.813066	0.183796
H	1.715146	-2.495396	0.287201
O	-0.791189	-0.118403	2.983444
O	0.937589	1.138664	2.301046
C	0.625801	3.284499	1.318296
C	-0.779682	3.125549	1.373509
C	1.446764	2.287338	1.743495
C	-1.359894	1.958269	1.885119
C	-0.436310	0.919018	2.432028
H	1.070248	4.184690	0.902357
O	-2.627651	1.727008	1.944451
H	-1.441360	3.907794	1.008728
H	2.529106	2.279697	1.721752
H	-3.211235	0.396292	2.384395

N	-3.976989	-0.332589	2.721430
C	-3.731069	-0.584604	4.191569
C	-3.913927	-1.602213	1.887739
C	-5.289444	0.387209	2.486780
H	-2.759872	-1.073472	4.247151
H	-4.505324	-1.278402	4.543991
C	-3.691044	0.694815	5.029121
C	-2.773338	-2.555465	2.234769
H	-3.799444	-1.270548	0.856158
H	-4.886474	-2.098808	2.000865
H	-6.085777	-0.245614	2.899431
C	-5.540064	0.731901	1.017796
H	-5.237896	1.308320	3.068100
H	-4.682956	1.273034	0.606599
H	-6.420509	1.381845	0.960750
H	-5.737620	-0.152393	0.405470
H	-3.311426	0.442995	6.025370
H	-4.676411	1.153559	5.155681
H	-3.013700	1.425274	4.577454
H	-2.732051	-3.320134	1.451739
H	-2.925271	-3.062720	3.192902
H	-1.818389	-2.029271	2.249928

catalyzed_TS$_{DA}$_*exo* (in gas)

E = -6254.22

H = -6000.35

G = -6049.11

N_{imag} = 0, ν = i454.88372 cm^{-1}

O	-1.842840	-0.942240	-0.295976
N	0.505954	-0.922582	-0.290181
O	2.723085	-0.233646	-0.511873
C	-0.786911	-0.302619	-0.363763
C	-0.554234	1.114098	-0.544913
C	0.838249	1.369228	-0.549846
C	1.522803	0.009714	-0.467296
C	0.724657	-2.326790	0.021907
H	-1.348101	1.794025	-0.817501
H	1.311753	2.119416	-1.176048
H	1.022501	-2.451128	1.070257
H	-0.214190	-2.856500	-0.155296
H	1.514422	-2.726168	-0.620783
O	-0.859943	-0.041519	2.936259
O	0.888312	1.077825	2.081271
C	0.624373	3.335266	1.268184
C	-0.712850	3.324460	1.574774
C	1.334815	2.100377	1.226144
C	-1.357586	2.091486	1.934856
C	-0.462820	0.947039	2.326880
H	1.121919	4.231836	0.906051
O	-2.607229	1.951233	2.096085
H	-1.329399	4.218314	1.511574

H	2.419927	2.079640	1.186682
H	-3.131232	0.334325	2.446184
N	-3.911228	-0.336941	2.729652
C	-3.794965	-0.509272	4.230958
C	-3.793333	-1.660440	1.979197
C	-5.185899	0.387083	2.333182
H	-2.826854	-0.981243	4.392902
H	-4.588160	-1.201424	4.538019
C	-3.852608	0.806723	5.006729
C	-2.678694	-2.589229	2.452838
H	-3.609350	-1.391075	0.940154
H	-4.774315	-2.143048	2.069628
H	-6.023011	-0.209607	2.714748
C	-5.295119	0.634097	0.828776
H	-5.163416	1.342602	2.856851
H	-4.391289	1.125712	0.458350
H	-6.146780	1.300051	0.650869
H	-5.461309	-0.284563	0.260334
H	-3.566817	0.605269	6.044921
H	-4.852616	1.250479	5.017980
H	-3.147281	1.529987	4.587727
H	-2.618759	-3.413911	1.734402
H	-2.873312	-3.018999	3.440750
H	-1.716753	-2.074227	2.460911

catalyzed_Product$_{DA}$_*exo* (in gas)
E = -6265.7
H = -6010.69
G = -6060.06
N_{imag} = 0

O	-1.588185	-1.361058	0.132356
N	0.691273	-1.063001	0.436354
O	2.865963	-0.228613	0.372890
C	-0.616346	-0.626661	0.159544
C	-0.568476	0.875706	-0.100232
C	0.935207	1.224456	-0.127391
C	1.664208	-0.072308	0.253695
C	1.000212	-2.445497	0.803358
H	-1.102262	1.098586	-1.026558
H	1.286778	1.550593	-1.111225
H	0.586872	-3.126395	0.053626
H	2.086382	-2.539720	0.852059
H	0.555887	-2.678486	1.775579
O	-0.961425	1.032744	3.376405
O	0.829147	1.784042	2.233212
C	0.297248	3.512709	0.595422
C	-0.999527	3.199609	0.668707
C	1.202453	2.347423	0.911111
C	-1.299834	1.751947	1.041416
C	-0.505153	1.481263	2.350443
H	0.697270	4.487648	0.335311

O	-2.661613	1.531426	1.133720
H	-1.838958	3.867010	0.499383
H	2.261627	2.585122	1.001149
H	-2.927297	0.736469	1.722771
N	-3.965048	-0.358626	2.604917
C	-4.103058	0.170459	3.991319
C	-3.554669	-1.790859	2.626217
C	-5.243839	-0.206470	1.854120
H	-3.145348	0.009812	4.487870
H	-4.868754	-0.415960	4.536097
C	-4.433199	1.665666	4.071751
C	-2.242505	-2.076063	3.364855
H	-3.436694	-2.103547	1.588417
H	-4.368747	-2.397137	3.070453
H	-6.047780	-0.762302	2.375176
C	-5.169726	-0.642949	0.385326
H	-5.510403	0.852825	1.886320
H	-4.311960	-0.181499	-0.112884
H	-6.084647	-0.323289	-0.127571
H	-5.090584	-1.728446	0.273101
H	-4.344188	1.992163	5.114444
H	-5.451970	1.894140	3.742068
H	-3.730293	2.248364	3.468877
H	-1.932032	-3.103958	3.143614
H	-2.338771	-1.985656	4.451165
H	-1.452227	-1.395396	3.039634

catalyzed_TS$_{retro\text{-}DA}$_*exo* (in gas)
E = -6245.83
H = -5992.73
G = -6042.92
N_{imag} = 0, ν = i490.79276 cm^{-1}

O	-1.543227	-1.401111	0.303532
N	0.740091	-1.022975	0.428796
O	2.876489	-0.115936	0.223378
C	-0.594855	-0.641073	0.226859
C	-0.617157	0.848475	-0.121792
C	0.869587	1.221706	-0.309154
C	1.666892	-0.013332	0.153259
C	1.117757	-2.364079	0.876625
H	-1.209059	0.978139	-1.032938
H	1.121231	1.370712	-1.370217
H	0.580237	-3.107645	0.282450
H	2.196543	-2.467267	0.748093
H	0.853532	-2.492679	1.931097
O	-1.057502	0.960531	3.364541
O	0.882213	1.558821	2.307925
C	0.320316	3.522193	0.534877
C	-0.990379	3.151320	0.775177
C	1.247630	2.479823	0.422548
C	-1.311360	1.753098	0.967089

C	-0.356575	1.390531	2.462746
H	0.651846	4.546949	0.678822
O	-2.643851	1.511314	1.158888
H	-1.759244	3.878223	1.027779
H	2.310718	2.655003	0.566313
H	-2.882278	0.698745	1.741190
N	-3.980750	-0.361749	2.603568
C	-4.146574	0.194585	3.976417
C	-3.615963	-1.805533	2.665781
C	-5.231761	-0.186902	1.812943
H	-3.209454	0.015853	4.504645
H	-4.944851	-0.362539	4.505162
C	-4.437820	1.698963	4.025881
C	-2.338241	-2.115850	3.453341
H	-3.474370	-2.142388	1.638806
H	-4.463832	-2.376431	3.093689
H	-6.067657	-0.706514	2.321146
C	-5.130325	-0.657860	0.356499
H	-5.466070	0.880451	1.814659
H	-4.245940	-0.232059	-0.126625
H	-6.020725	-0.323375	-0.189160
H	-5.079771	-1.747399	0.269825
H	-4.383708	2.034664	5.068157
H	-5.435197	1.951760	3.651199
H	-3.692657	2.252653	3.447644
H	-2.046064	-3.153451	3.253458
H	-2.472399	-2.011595	4.534553
H	-1.521355	-1.457715	3.148869

catalyzed_Product$_{retro\text{-}DA}$_*exo* (in gas)

E = -5773.33

H = -5529.53

G = -5575.93

N_{imag} = 0

O	-1.161598	-1.660619	0.254594
N	1.002656	-1.066914	-0.310312
O	2.955269	0.087095	-0.829181
C	-0.344775	-0.805368	-0.040978
C	-0.560133	0.722225	-0.217192
C	0.881080	1.304612	-0.241224
C	1.775750	0.085110	-0.522817
C	1.549140	-2.422792	-0.344772
H	-1.045814	0.841951	-1.194961
H	1.032917	2.026417	-1.055455
H	0.997245	-3.028556	-1.069801
H	2.599603	-2.346838	-0.631756
H	1.457527	-2.887673	0.642135
H	-1.606988	-2.061316	3.780557
H	-1.464134	-0.424062	3.121872
C	0.416311	2.164887	2.058998
C	-1.011126	1.909469	1.925077

C	1.309574	1.912686	1.082211
C	-1.499719	1.261800	0.838227
H	-2.192541	-0.687249	4.723564
H	0.763479	2.615583	2.987868
O	-2.792663	0.969136	0.594109
H	-1.689373	2.268713	2.694393
H	2.365154	2.143955	1.193347
H	-3.301963	0.696281	1.462026
N	-4.164031	-0.026660	2.633740
C	-4.366426	0.883604	3.792990
C	-3.462748	-1.287272	3.027094
C	-5.466574	-0.343754	1.974655
H	-3.396719	1.001904	4.281859
H	-5.045495	0.412622	4.528196
C	-4.887228	2.272933	3.402745
C	-2.105310	-1.088236	3.708355
H	-3.297667	-1.851748	2.107407
H	-4.128545	-1.884384	3.677523
H	-6.129163	-0.847852	2.702685
C	-5.339961	-1.188079	0.699897
H	-5.935522	0.609277	1.716236
H	-4.599829	-0.759044	0.017483
H	-6.311017	-1.209558	0.191828
H	-5.055224	-2.223116	0.910447
H	-4.843976	2.933867	4.275995
H	-5.925744	2.252880	3.058586
H	-4.271260	2.712320	2.610082

uncatalyzed_RC_*endo* (in chloroform)
E = -3645.3
H = -3525.42
G = -3562.3
N_{imag} = 0

O	2.588837	0.190646	1.674377
N	0.266527	0.021441	1.900693
O	-1.915752	-0.784338	1.699468
C	1.523869	-0.352463	1.402959
C	1.281603	-1.527162	0.496356
C	-0.029310	-1.810089	0.498063
C	-0.727073	-0.841051	1.409663
C	0.020724	1.118905	2.833015
H	2.088360	-2.021021	-0.030624
H	-0.562054	-2.593602	-0.026483
H	-0.308081	0.732474	3.802706
H	-0.750425	1.782637	2.431810
H	0.955811	1.668134	2.955367
O	-1.371613	-1.611907	-3.012670
O	0.623263	-0.611193	-2.642875
C	0.786777	1.330845	-1.268310
C	-0.621730	1.318721	-1.062181
C	1.363507	0.371641	-2.035628

C	-1.369491	0.326463	-1.638510
C	-0.752666	-0.699047	-2.468385
H	1.411346	2.090647	-0.811898
O	-2.717519	0.240410	-1.485991
H	-1.103395	2.077440	-0.452188
H	2.417619	0.280760	-2.264716
H	-3.011362	-0.549188	-1.991826

uncatalyzed_TS$_{DA}$_*endo* (in chloroform)
E = -3628.95
H = -3509.18
G = -3544.09
N_{imag} = 0, ν = i456.423041 cm^{-1}

O	2.592522	0.234060	1.536079
N	0.276837	0.121131	1.820934
O	-1.926459	-0.662572	1.759691
C	1.488364	-0.187854	1.205633
C	1.149686	-1.098674	0.043184
C	-0.227374	-1.414070	0.165622
C	-0.788260	-0.661177	1.293760
C	0.123333	1.029822	2.954317
H	1.888231	-1.838873	-0.249380
H	-0.729911	-2.281208	-0.240620
H	-0.152608	0.470516	3.853884
H	-0.658343	1.763121	2.737453
H	1.077680	1.534462	3.112658
O	-1.279220	-1.659625	-3.098144
O	0.707850	-0.769418	-2.483160
C	0.735220	1.263487	-1.156879
C	-0.638470	1.295266	-1.129060
C	1.363594	0.035233	-1.520938
C	-1.318715	0.131914	-1.538743
C	-0.660702	-0.846362	-2.417130
H	1.342029	2.074162	-0.768987
O	-2.657781	0.050557	-1.450682
H	-1.201869	2.142472	-0.750052
H	2.431583	0.008291	-1.705423
H	-2.938521	-0.734944	-1.974568

uncatalyzed_Product$_{DA}$_*endo* (in chloroform)
E = -3649.41
H = -3527.83
G = -3562.03
N_{imag} = 0

O	2.529451	0.152784	1.574219
N	0.228157	0.216004	1.911652
O	-2.066606	-0.034183	1.732134
C	1.390842	-0.125278	1.223291
C	0.986279	-0.881045	-0.041947
C	-0.556025	-0.947769	-0.002862
C	-0.945969	-0.221043	1.285687

C	0.215902	0.954127	3.177745
H	1.458138	-1.866930	-0.040617
H	-0.943702	-1.968805	0.028376
H	-0.308412	0.368137	3.936958
H	-0.297552	1.910267	3.043179
H	1.250954	1.122280	3.476545
O	-1.091194	-1.636753	-3.301691
O	0.884856	-0.940299	-2.459023
C	0.750006	1.240937	-1.355615
C	-0.585295	1.176596	-1.346933
C	1.415389	-0.112082	-1.327156
C	-1.135135	-0.239180	-1.304998
C	-0.464572	-1.008847	-2.466269
H	1.336515	2.153074	-1.364465
O	-2.538647	-0.311764	-1.397454
H	-1.272960	2.015793	-1.366084
H	2.492710	-0.098939	-1.470643
H	-2.734601	-0.919271	-2.142799

uncatalyzed_TS$_{retro\text{-}DA}$_*endo* (in chloroform)
E = -3633.09
H = -3513.28
G = -3547.87
N_{imag} = 0, ν = i334.854144 cm^{-1}

O	2.532752	0.157274	1.799543
N	0.223543	0.166570	2.006251
O	-2.055531	-0.151358	1.707220
C	1.420477	-0.068790	1.348967
C	1.088920	-0.646590	-0.050672
C	-0.444677	-0.863550	-0.035241
C	-0.913417	-0.250813	1.295661
C	0.166513	0.731738	3.359106
H	1.656159	-1.571574	-0.172997
H	-0.704096	-1.925398	-0.020008
H	-0.882965	0.891715	3.607464
H	0.712446	1.677976	3.379344
H	0.620048	0.036235	4.070854
O	-1.294528	-1.777038	-3.187025
O	0.823369	-1.069584	-2.677583
C	0.665947	1.413393	-1.454779
C	-0.689706	1.147515	-1.511040
C	1.507304	0.360793	-1.084288
C	-1.130009	-0.210098	-1.269422
C	-0.418859	-1.140159	-2.588402
H	1.077611	2.326427	-1.874417
O	-2.508908	-0.419702	-1.342088
H	-1.401624	1.865295	-1.909767
H	2.565305	0.400143	-1.327028
H	-2.614228	-1.083365	-2.070957

uncatalyzed_Product$_{retro\text{-}DA}$_*endo* (in chloroform)

E = -3154.53
H = -3043.36
G = -3075.07
N_{imag} = 0

O	2.435440	0.330709	1.807981
N	0.137589	0.084713	2.049323
O	-2.116851	-0.340287	1.696905
C	1.352449	0.013612	1.344793
C	1.064862	-0.543047	-0.064199
C	-0.482322	-0.692137	-0.108055
C	-0.950250	-0.320002	1.302781
C	0.026941	0.536680	3.440411
H	1.535819	-1.535364	-0.078009
H	-0.790238	-1.731320	-0.285243
H	-0.395934	-0.262818	4.054590
H	-0.622657	1.414530	3.490875
H	1.029195	0.789248	3.787637
H	-1.112642	1.546127	-2.720081
H	2.760604	0.358897	-1.186678
C	0.909916	0.986906	-2.027586
C	-0.548762	0.952096	-2.005247
C	1.676251	0.311420	-1.151503
C	-1.206601	0.186635	-1.113395
H	-2.848244	-0.197943	-0.186270
H	1.384690	1.595239	-2.795070
O	-2.578671	0.119659	-1.079016

uncatalyzed_RC_*exo* (in chloroform)
E = -3644.63
H = -3524.18
G = -3563.53
N_{imag} = 0

O	-1.950034	-0.257451	-1.144839
N	0.237410	-0.800523	-0.533430
O	2.532208	-0.593306	-0.125090
C	-0.774834	0.054215	-0.993795
C	-0.123172	1.386480	-1.243391
C	1.180259	1.285638	-0.950933
C	1.461919	-0.117168	-0.485290
C	0.030444	-2.193596	-0.139705
H	-0.682513	2.239868	-1.605888
H	1.958813	2.035385	-1.014874
H	-0.448876	-2.742273	-0.954989
H	1.009092	-2.626780	0.073587
H	-0.598492	-2.238403	0.753997
O	-1.597542	-0.458298	2.344391
O	0.358167	0.669831	2.486340
C	0.471585	3.000844	2.001535
C	-0.918571	3.013618	1.688482
C	1.066393	1.841899	2.376050
C	-1.632595	1.848959	1.778446

C	-1.004593	0.611081	2.215750
H	1.067279	3.904356	1.930317
O	-2.960311	1.773860	1.487938
H	-1.410452	3.929966	1.374278
H	2.110589	1.702810	2.625627
H	-3.225221	0.835785	1.612862

uncatalyzed_TS_{DA}_*exo* (in chloroform)
E = -3627.57
H = -3507.87
G = -3543.19
N_{imag} = 0, ν = i479.331595 cm^{-1}

O	-1.900255	-0.527504	-1.083682
N	0.322402	-0.903186	-0.436125
O	2.572066	-0.558550	0.050344
C	-0.804492	-0.088912	-0.740721
C	-0.373858	1.304261	-0.562632
C	0.984100	1.322578	-0.161868
C	1.443325	-0.129428	-0.167023
C	0.303058	-2.363653	-0.398778
H	-0.930975	2.118721	-1.003213
H	1.694686	2.067281	-0.503763
H	-0.691335	-2.688796	-0.708599
H	1.056570	-2.764814	-1.082526
H	0.509840	-2.719029	0.615424
O	-1.873016	-0.207792	2.278664
O	0.213485	0.670766	2.266036
C	0.505896	3.034998	1.823155
C	-0.851067	3.198749	1.671386
C	1.027792	1.710949	1.769450
C	-1.629919	2.032455	1.530833
C	-1.139086	0.744895	2.031763
H	1.194371	3.873639	1.820485
O	-2.947347	2.102140	1.265314
H	-1.321026	4.173604	1.582622
H	2.060520	1.506810	2.030250
H	-3.318205	1.199660	1.404696

uncatalyzed_$Product_{DA}$_*exo* (in chloroform)
E = -3649.54
H = -3527.95
G = -3561.88
N_{imag} = 0

O	-2.073994	-0.715117	0.434956
N	0.086871	-0.919913	-0.391095
O	2.276463	-0.505156	-1.064869
C	-1.008040	-0.211769	0.094210
C	-0.634222	1.262772	0.188569
C	0.830846	1.333922	-0.282778
C	1.202629	-0.108016	-0.643237
C	0.073765	-2.374499	-0.580220

H	-1.325063	1.860070	-0.410276
H	0.973945	1.967320	-1.161505
H	-0.704078	-2.643524	-1.299533
H	1.053795	-2.670284	-0.955204
H	-0.129965	-2.866405	0.374518
O	-0.080697	0.140601	3.414559
O	1.507766	0.933975	2.026377
C	1.122733	3.234034	1.291592
C	-0.143987	3.174006	1.713133
C	1.685158	1.892752	0.889883
C	-0.741833	1.780277	1.686431
C	0.226646	0.872409	2.497233
H	1.720063	4.136268	1.220204
O	-2.060826	1.769065	2.178211
H	-0.750353	4.003920	2.061638
H	2.752461	1.877208	0.681296
H	-2.437249	0.879591	2.015256

uncatalyzed_$TS_{retro\text{-}DA}$_*exo* (in chloroform)
E = -3633.35
H = -3513.7
G = -3547.73
N_{imag} = 0, ν = i288.555164 cm^{-1}

O	-2.078382	-0.806504	0.280338
N	0.131934	-0.873380	-0.432495
O	2.293413	-0.331091	-1.079320
C	-1.029045	-0.237024	0.031334
C	-0.732542	1.260595	0.167339
C	0.684670	1.426482	-0.421871
C	1.185064	-0.004003	-0.687010
C	0.244653	-2.324142	-0.614448
H	-1.491402	1.818938	-0.386312
H	0.657756	1.923666	-1.403291
H	-0.751498	-2.752801	-0.502298
H	0.642006	-2.534542	-1.610031
H	0.917791	-2.740358	0.140825
O	-0.398636	0.041814	3.368782
O	1.412823	0.621724	2.094236
C	1.094630	3.260807	1.239007
C	-0.151711	3.063029	1.803114
C	1.599351	2.237557	0.438808
C	-0.791160	1.768172	1.652809
C	0.234118	0.672919	2.510579
H	1.732319	4.080240	1.557499
O	-2.067273	1.682547	2.219016
H	-0.577027	3.762279	2.518425
H	2.664839	2.158721	0.238628
H	-2.004234	0.927712	2.855376

uncatalyzed_$Product_{retro\text{-}DA}$_*exo* (in chloroform)
E = -3154.53

H = -3043.36
G = -3074.95
N_{imag} = 0

O	-2.103249	-0.615968	0.432966
N	0.102842	-0.830890	-0.255791
O	2.285388	-0.424984	-0.941343
C	-1.032100	-0.121227	0.080117
C	-0.732310	1.378872	-0.008148
C	0.725886	1.460561	-0.541989
C	1.185843	-0.009398	-0.615281
C	0.160278	-2.296523	-0.243669
H	-1.453511	1.809829	-0.715399
H	0.723190	1.809082	-1.583701
H	-0.547881	-2.700274	-0.972507
H	1.177210	-2.590711	-0.504777
H	-0.096576	-2.664154	0.753091
H	-2.651789	1.061371	1.428187
H	-0.263415	3.161095	2.964053
C	1.301032	2.875631	1.429697
C	-0.030705	2.709832	2.002790
C	1.676545	2.315924	0.264869
C	-0.979105	2.009414	1.351613
H	2.676426	2.450682	-0.136894
H	2.013416	3.482457	1.985393
O	-2.246845	1.850252	1.857366

catalyzed_RC_*endo* (in chloroform)
E = -6275.8
H = -6021.39
G = -6075.42
N_{imag} = 0

O	2.319045	0.866154	2.522750
N	0.148858	0.208881	1.947179
O	-1.628869	-1.017188	1.046441
C	1.545429	0.129399	1.910120
C	1.871271	-1.017425	1.003086
C	0.719628	-1.561973	0.562255
C	-0.423183	-0.809908	1.155003
C	-0.622574	1.213549	2.668850
H	2.890588	-1.325532	0.810306
H	0.581530	-2.417788	-0.084547
H	0.081719	1.914970	3.120099
H	-1.227640	0.746428	3.452623
H	-1.283154	1.744399	1.976504
O	-0.086991	-1.428834	-3.028261
O	1.753904	-0.421605	-2.224840
C	1.642192	1.561623	-0.912262
C	0.233784	1.568954	-1.011946
C	2.366679	0.566234	-1.497070
C	-0.460358	0.566539	-1.703494
C	0.355711	-0.500531	-2.357308

H	2.168233	2.331249	-0.355456
O	-1.741709	0.517708	-1.828365
H	-0.346190	2.358406	-0.539932
H	3.444584	0.465305	-1.494469
H	-2.587461	-0.787887	-2.087848
H	-2.396444	-0.186022	-4.453967
H	-3.136169	-1.638899	-5.168567
H	-4.512321	0.146590	-3.073117
H	-5.076372	-1.454325	-3.588143
H	-5.104297	-2.140721	-1.168655
H	-3.611017	-1.914895	-0.225171
H	-5.134003	-0.196180	0.480198
H	-5.456091	0.356554	-1.167392
H	-3.786394	-3.420590	-2.946708
H	-2.248435	-2.693043	-3.470476
H	-1.649349	-4.407402	-1.917262
H	-1.408206	-2.872228	-1.066709
C	-3.344781	-0.681762	-4.682867
C	-4.188382	-0.835717	-3.417311
N	-3.426530	-1.455470	-2.261056
C	-4.252801	-1.475226	-0.987100
C	-4.696950	-0.090184	-0.518638
C	-2.916855	-2.837048	-2.623648
C	-2.161183	-3.535757	-1.495990
H	-2.820217	-3.885581	-0.696884
H	-3.899835	-0.059876	-5.393333
H	-3.835386	0.580056	-0.448240

catalyzed_TS$_{DA}$_*endo* (in chloroform)
E = -6266.09
H = -6011.95
G = -6063.73
N_{imag} = 0, ν = i438.37968 cm^{-1}

O	2.451598	0.733121	2.429982
N	0.189277	0.386747	2.003862
O	-1.833559	-0.604308	1.300944
C	1.540416	0.174155	1.809897
C	1.655570	-0.830463	0.681818
C	0.337332	-1.278359	0.414265
C	-0.601923	-0.529984	1.211672
C	-0.370161	1.362752	2.928867
H	2.513637	-1.496340	0.688658
H	0.056950	-2.166289	-0.130990
H	-0.017942	2.369168	2.679333
H	-0.079802	1.131670	3.959535
H	-1.457234	1.317256	2.835604
O	0.037179	-1.480840	-3.009879
O	1.793781	-0.585248	-1.927025
C	1.570687	1.488298	-0.706911
C	0.257648	1.601679	-1.077209
C	2.233973	0.235940	-0.871257

C	-0.417102	0.501118	-1.699327
C	0.436832	-0.621767	-2.229439
H	2.082801	2.275044	-0.161123
O	-1.662026	0.493396	-1.935948
H	-0.322427	2.497856	-0.871076
H	3.316273	0.190230	-0.806642
H	-2.534675	-0.878669	-2.210606
H	-2.448062	-0.218128	-4.569382
H	-3.264048	-1.639225	-5.267047
H	-4.471782	0.137108	-3.061809
H	-5.108200	-1.437737	-3.573498
H	-4.946760	-2.252660	-1.125070
H	-3.409442	-1.914484	-0.285435
H	-4.973648	-0.276000	0.480429
H	-5.448351	0.213774	-1.151623
H	-3.842832	-3.443750	-3.051055
H	-2.316893	-2.756525	-3.652676
H	-1.765611	-4.576804	-2.162692
H	-1.276662	-3.069347	-1.385053
C	-3.419985	-0.688095	-4.751134
C	-4.196366	-0.845426	-3.444026
N	-3.385297	-1.510818	-2.345273
C	-4.125850	-1.536158	-1.014612
C	-4.618714	-0.167882	-0.549747
C	-2.935941	-2.898833	-2.767831
C	-2.140405	-3.653619	-1.708112
H	-2.741161	-3.927922	-0.837419
H	-3.997028	-0.037568	-5.416744
H	-3.797822	0.554281	-0.547073

catalyzed_Product$_{DA}$_*endo* (in chloroform)

E = -6274.85

H = -6020.64

G = -6069.16

N_{imag} = 0

O	2.250300	0.268016	2.766453
N	0.060123	0.761338	2.188306
O	-2.016934	0.876949	1.157062
C	1.293630	0.147946	2.010165
C	1.242188	-0.659331	0.716209
C	-0.188652	-0.466389	0.171465
C	-0.871247	0.458608	1.179333
C	-0.235946	1.630766	3.329621
H	1.488179	-1.703436	0.927339
H	-0.754094	-1.398878	0.111812
H	-1.288881	1.908385	3.272496
H	0.391078	2.526080	3.286850
H	-0.034140	1.094651	4.260559
O	0.486850	-1.407532	-3.104755
O	2.040526	-0.953015	-1.543092
C	1.895771	1.323536	-0.672598

C	0.667325	1.464247	-1.180414
C	2.247038	-0.105113	-0.337266
C	-0.123018	0.170645	-1.299397
C	0.797942	-0.808393	-2.096585
H	2.596530	2.130808	-0.489063
O	-1.359255	0.368970	-1.894380
H	0.203798	2.393871	-1.495578
H	3.283322	-0.264922	-0.048310
H	-1.858237	-0.517259	-2.031969
N	-2.966557	-1.791589	-2.376051
C	-3.930201	-1.928682	-1.244528
C	-3.660998	-1.245865	-3.581878
C	-2.348891	-3.109898	-2.700873
C	-4.532109	-0.605610	-0.757976
H	-3.388322	-2.384566	-0.411886
H	-4.734161	-2.632884	-1.530373
C	-2.736245	-0.967367	-4.773299
H	-4.131944	-0.306702	-3.282456
H	-4.471771	-1.935829	-3.881768
H	-1.683016	-2.950619	-3.550065
H	-3.140161	-3.812666	-3.023090
C	-1.533761	-3.743094	-1.569312
H	-1.049888	-4.650241	-1.948805
H	-2.144458	-4.031106	-0.708509
H	-0.741031	-3.070149	-1.228909
H	-1.869049	-0.376769	-4.462029
H	-3.293027	-0.399840	-5.528022
H	-2.376082	-1.884422	-5.249482
H	-3.744133	0.111460	-0.509762
H	-5.113915	-0.797314	0.151438
H	-5.209371	-0.156584	-1.491081

catalyzed_Product$_{DA}$′_*endo* (in chloroform)
E = -3649.39
H = -3527.8
G = -3561.71
N_{imag} = 0

O	2.349166	0.425613	2.716568
N	0.105718	0.758604	2.233975
O	-2.014591	0.727645	1.284433
C	1.371184	0.227143	2.007685
C	1.316398	-0.612054	0.732454
C	-0.145195	-0.510587	0.244428
C	-0.843915	0.384420	1.270886
C	-0.197616	1.620201	3.380221
H	1.622122	-1.636741	0.958924
H	-0.655552	-1.475929	0.205254
H	-1.265828	1.837763	3.359889
H	0.376798	2.548080	3.308165
H	0.067523	1.103330	4.306069
O	0.358464	-1.486069	-2.985954

O	2.017544	-0.916921	-1.563509
C	1.821394	1.371173	-0.715186
C	0.563419	1.450274	-1.160700
C	2.248765	-0.034059	-0.373510
C	-0.156221	0.113365	-1.219794
C	0.751257	-0.842466	-2.028650
H	2.495948	2.209646	-0.580835
O	-1.444731	0.181136	-1.784213
H	0.042001	2.350859	-1.468254
H	3.303670	-0.150753	-0.139069
H	-1.462580	-0.478961	-2.510134

catalyzed_$TS_{retro\text{-}DA}$_*endo* (in chloroform)
E = -6256.55
H = -6004.02
G = -6053.07
N_{imag} = 0, ν = i475.384846 cm^{-1}

O	1.988279	0.065246	2.984872
N	-0.090745	0.717646	2.193820
O	-2.027155	1.003912	0.952692
C	1.139624	0.086729	2.104474
C	1.247226	-0.544849	0.700298
C	-0.153806	-0.364790	0.069777
C	-0.909725	0.532619	1.067437
C	-0.514611	1.461677	3.384177
H	1.542687	-1.589508	0.816923
H	-0.694505	-1.312179	0.003698
H	-1.457016	1.956796	3.148129
H	0.250014	2.198504	3.641498
H	-0.652051	0.774784	4.224331
O	0.403320	-1.360754	-3.251794
O	2.066549	-1.136668	-1.705348
C	1.980411	1.399885	-0.747592
C	0.756103	1.450453	-1.385236
C	2.297519	0.208388	-0.077521
C	-0.088690	0.273903	-1.351178
C	0.957756	-0.908603	-2.256949
H	2.750027	2.139704	-0.946339
O	-1.287760	0.400413	-1.999141
H	0.488345	2.265021	-2.053770
H	3.331624	-0.018551	0.163881
H	-1.798624	-0.493611	-2.056484
N	-2.911473	-1.768329	-2.303722
C	-3.806596	-1.913873	-1.117605
C	-3.683096	-1.250381	-3.475180
C	-2.289815	-3.081007	-2.647884
C	-4.501004	-0.620651	-0.677495
H	-3.188948	-2.273506	-0.289988
H	-4.559759	-2.697880	-1.321897
C	-2.839620	-1.015404	-4.734398
H	-4.124224	-0.298387	-3.171154

H	-4.517823	-1.940535	-3.698962
H	-1.659009	-2.916684	-3.521785
H	-3.086431	-3.793021	-2.934236
C	-1.424583	-3.700929	-1.546996
H	-0.953569	-4.608730	-1.940838
H	-1.996836	-3.985153	-0.658893
H	-0.619558	-3.023029	-1.247481
H	-1.937953	-0.442848	-4.496391
H	-3.436094	-0.447100	-5.457552
H	-2.537273	-1.949632	-5.217375
H	-3.774787	0.186620	-0.547916
H	-4.986056	-0.794504	0.290260
H	-5.276016	-0.300961	-1.380689

catalyzed_Product$_{retro\text{-}DA}$_*endo* (in chloroform)

E = -5780.99

H = -5537.52

G = -5584.42

N_{imag} = 0

O	-0.464035	-1.991961	-0.050192
N	1.462782	-0.831074	-0.586916
O	3.034923	0.813581	-1.059936
C	0.114690	-0.932231	-0.235718
C	-0.463756	0.500864	-0.174122
C	0.794719	1.414383	-0.204042
C	1.915029	0.484764	-0.688429
C	2.298219	-2.005932	-0.846528
H	-1.043204	0.622867	-1.098203
H	0.701838	2.234369	-0.928246
H	1.866442	-2.596419	-1.659464
H	3.292142	-1.653552	-1.124659
H	2.355326	-2.623743	0.054143
H	-2.471482	-2.389448	4.454585
H	-1.962861	-1.430561	3.050173
C	0.357575	1.856276	2.215467
C	-0.976122	1.279922	2.137448
C	1.189607	1.954380	1.159178
C	-1.406364	0.678036	0.998343
H	-2.279066	-0.640351	4.607782
H	0.671984	2.263669	3.175552
O	-2.625555	0.157511	0.765726
H	-1.631711	1.367109	2.996323
H	2.164066	2.428668	1.236283
H	-3.249582	0.141050	1.604776
N	-4.389531	-0.047487	2.767477
C	-4.391290	1.134268	3.680006
C	-4.082923	-1.294737	3.533830
C	-5.710295	-0.175255	2.077121
H	-3.485836	1.073485	4.290318
H	-5.247057	1.061138	4.373862
C	-4.411058	2.483683	2.952328

C	-2.610376	-1.435750	3.932452
H	-4.351828	-2.141748	2.898459
H	-4.731713	-1.340547	4.425888
H	-6.476421	-0.478819	2.812126
C	-5.700335	-1.139345	0.885258
H	-5.987238	0.818510	1.717457
H	-4.948441	-0.842514	0.147336
H	-6.684560	-1.120230	0.403101
H	-5.500144	-2.173748	1.182591
H	-4.290131	3.288605	3.686305
H	-5.352683	2.659993	2.423496
H	-3.590905	2.556590	2.230474

catalyzed_RC_*exo* (in chloroform)

E = -6274.59

H = -6020.69

G = -6072.73

N_{imag} = 0

O	-1.790078	-0.682149	-0.414203
N	0.535913	-0.825274	-0.189402
O	2.810265	-0.325317	-0.388418
C	-0.671146	-0.195899	-0.553077
C	-0.289027	1.118706	-1.153252
C	1.054175	1.217149	-1.157943
C	1.628295	-0.021627	-0.547439
C	0.639035	-2.119617	0.475355
H	-1.029079	1.818487	-1.518369
H	1.680269	2.015874	-1.533488
H	0.658166	-1.991703	1.563025
H	-0.227962	-2.722232	0.198028
H	1.557711	-2.611739	0.148533
O	-0.737089	-0.066780	3.019343
O	0.972426	1.204370	2.320995
C	0.638027	3.318354	1.276512
C	-0.763962	3.135893	1.320472
C	1.472253	2.348974	1.737813
C	-1.336710	1.976082	1.864981
C	-0.398588	0.964275	2.441866
H	1.070981	4.212780	0.837243
O	-2.598487	1.733794	1.932999
H	-1.430669	3.899063	0.925259
H	2.554396	2.364117	1.738184
H	-3.233682	0.339639	2.415136
N	-3.994956	-0.357158	2.743185
C	-3.769433	-0.607544	4.220471
C	-3.925590	-1.632028	1.915656
C	-5.308399	0.364901	2.494578
H	-2.812909	-1.122082	4.292006
H	-4.563540	-1.281302	4.561196
C	-3.713277	0.674603	5.050845
C	-2.753072	-2.551741	2.243810

H	-3.848174	-1.303798	0.879644
H	-4.882616	-2.146583	2.056930
H	-6.102559	-0.266314	2.908258
C	-5.551651	0.694696	1.021901
H	-5.259819	1.287978	3.072183
H	-4.686950	1.215943	0.600152
H	-6.420985	1.358212	0.958869
H	-5.766111	-0.194163	0.422275
H	-3.373697	0.414138	6.059048
H	-4.686694	1.164361	5.144167
H	-2.998192	1.381009	4.618547
H	-2.710656	-3.324147	1.468510
H	-2.870289	-3.053009	3.209173
H	-1.811269	-2.001327	2.233635

catalyzed_TS_{DA}_*exo* (in chloroform)
E = -6265.44
H = -6011.73
G = -6061.16
N_{imag} = 0, ν = i450.58493 cm^{-1}

O	-1.763386	-0.981250	-0.357224
N	0.586767	-0.932222	-0.251096
O	2.798881	-0.235538	-0.437141
C	-0.714660	-0.323780	-0.390304
C	-0.489014	1.085860	-0.590106
C	0.901574	1.351090	-0.566705
C	1.587338	0.001932	-0.429014
C	0.808649	-2.336489	0.066735
H	-1.280162	1.760738	-0.882869
H	1.387084	2.095424	-1.189720
H	1.316363	-2.435853	1.032293
H	-0.169366	-2.819152	0.115160
H	1.418345	-2.813417	-0.707436
O	-0.854276	0.001502	2.928096
O	0.891422	1.126763	2.082527
C	0.620623	3.361418	1.208991
C	-0.718597	3.348208	1.503037
C	1.349734	2.138441	1.219664
C	-1.361861	2.123050	1.894558
C	-0.463770	0.987070	2.307053
H	1.114073	4.251087	0.826277
O	-2.608926	1.981741	2.052168
H	-1.336779	4.237982	1.407544
H	2.434880	2.134998	1.207654
H	-3.175871	0.312698	2.464862
N	-3.955762	-0.342185	2.744853
C	-3.834116	-0.549621	4.244048
C	-3.851676	-1.649769	1.963525
C	-5.237098	0.393966	2.380814
H	-2.888598	-1.067934	4.394105
H	-4.653895	-1.212199	4.540326

C	-3.832973	0.753197	5.041869
C	-2.720982	-2.580637	2.391372
H	-3.700611	-1.357062	0.925560
H	-4.826289	-2.138252	2.067007
H	-6.064903	-0.211804	2.763105
C	-5.373794	0.669818	0.884535
H	-5.208559	1.335988	2.927435
H	-4.478209	1.168069	0.501651
H	-6.227786	1.340007	0.738976
H	-5.557642	-0.237910	0.304059
H	-3.574912	0.515450	6.079349
H	-4.807399	1.249322	5.046953
H	-3.082008	1.445897	4.650357
H	-2.680576	-3.396629	1.661864
H	-2.887571	-3.022915	3.377996
H	-1.759835	-2.063962	2.378287

catalyzed_Product$_{DA}$_*exo* (in chloroform)

E = -6273.92

H = -6019.29

G = -6068.51

N_{imag} = 0

O	-1.601213	-1.355722	0.087107
N	0.672175	-1.067104	0.444216
O	2.847824	-0.247465	0.415270
C	-0.626401	-0.623085	0.143203
C	-0.571778	0.878880	-0.098019
C	0.934334	1.217025	-0.121057
C	1.644116	-0.082549	0.272638
C	0.974277	-2.460266	0.783882
H	-1.096466	1.113013	-1.025897
H	1.287884	1.525938	-1.108641
H	0.961234	-3.081425	-0.117329
H	1.965372	-2.490919	1.238020
H	0.220316	-2.824950	1.483728
O	-0.955498	1.073204	3.392524
O	0.829753	1.798515	2.234303
C	0.300500	3.513441	0.579627
C	-0.995917	3.203567	0.662336
C	1.207295	2.352035	0.898673
C	-1.296111	1.756227	1.044917
C	-0.500446	1.501784	2.352199
H	0.703094	4.482742	0.305649
O	-2.660781	1.536403	1.147326
H	-1.833138	3.872331	0.488714
H	2.264100	2.593939	0.989308
H	-2.928729	0.738277	1.733923
N	-3.962005	-0.357310	2.612184
C	-4.111874	0.167587	3.999814
C	-3.542606	-1.787697	2.636209
C	-5.244499	-0.217167	1.862476

H	-3.163877	-0.008046	4.509794
H	-4.890611	-0.412476	4.531226
C	-4.426182	1.665998	4.082804
C	-2.216164	-2.060141	3.353170
H	-3.441092	-2.106497	1.598475
H	-4.344986	-2.395840	3.096948
H	-6.039966	-0.785359	2.381416
C	-5.167079	-0.646458	0.391761
H	-5.525697	0.838040	1.900838
H	-4.310052	-0.179061	-0.102959
H	-6.083050	-0.328721	-0.120182
H	-5.082571	-1.731280	0.275508
H	-4.365448	1.982654	5.130706
H	-5.431470	1.909238	3.723911
H	-3.698191	2.244026	3.505268
H	-1.910369	-3.092339	3.146465
H	-2.290555	-1.948862	4.439305
H	-1.431818	-1.387595	2.997339

catalyzed_Product$_{DA}$′_*exo* (in chloroform)
E = -3649.53
H = -3527.95
G = -3561.75
N_{imag} = 0

O	-1.637601	-1.247998	0.471488
N	0.676752	-1.060829	0.422806
O	2.857016	-0.256725	0.280864
C	-0.622938	-0.585229	0.279544
C	-0.565294	0.893086	-0.087089
C	0.936082	1.237403	-0.120750
C	1.652564	-0.076935	0.205655
C	0.980637	-2.445619	0.799259
H	-1.082908	1.061415	-1.033859
H	1.277734	1.595057	-1.095008
H	0.547309	-3.127869	0.063519
H	2.065212	-2.553800	0.823138
H	0.558181	-2.659680	1.784672
O	-1.036952	0.985810	3.338830
O	0.787309	1.751795	2.259317
C	0.310844	3.519183	0.638268
C	-0.992659	3.227403	0.678663
C	1.199265	2.338732	0.944744
C	-1.294615	1.778720	1.011560
C	-0.546936	1.468989	2.339875
H	0.731082	4.490868	0.403451
O	-2.679754	1.537427	1.080961
H	-1.815383	3.913602	0.505074
H	2.255644	2.567886	1.065108
H	-2.815060	0.571135	1.166662

catalyzed_TS$_{retro\text{-}DA}$_*exo* (in chloroform)

E = -6255.21
H = -6002.52
G = -6052.4
N_{imag} = 0, ν = i407.349416 cm^{-1}

O	-1.538426	-1.402965	0.290408
N	0.741874	-1.022370	0.424398
O	2.875464	-0.112170	0.235781
C	-0.590520	-0.638137	0.221296
C	-0.616414	0.852139	-0.107606
C	0.867715	1.216299	-0.321903
C	1.661374	-0.013307	0.151518
C	1.114261	-2.377814	0.839095
H	-1.221633	0.996925	-1.006122
H	1.101032	1.331468	-1.391287
H	0.859126	-3.091927	0.050763
H	2.189216	-2.389208	1.021156
H	0.571572	-2.639265	1.750681
O	-1.072184	1.006512	3.402313
O	0.880723	1.530888	2.342728
C	0.337773	3.520249	0.520507
C	-0.965724	3.150016	0.796808
C	1.265034	2.492404	0.352866
C	-1.284549	1.748839	1.006202
C	-0.362910	1.397200	2.478291
H	0.667961	4.547495	0.645198
O	-2.629265	1.520781	1.188028
H	-1.725779	3.882446	1.058172
H	2.329685	2.677738	0.467680
H	-2.876872	0.712646	1.769569
N	-3.990467	-0.361584	2.617384
C	-4.185072	0.191098	3.987993
C	-3.613841	-1.802089	2.686718
C	-5.234817	-0.201030	1.811669
H	-3.263731	0.001057	4.539755
H	-4.999993	-0.360750	4.495504
C	-4.464702	1.697829	4.035155
C	-2.325524	-2.096342	3.462801
H	-3.479652	-2.145174	1.660777
H	-4.452383	-2.377548	3.125614
H	-6.068268	-0.737317	2.305194
C	-5.108193	-0.660388	0.353414
H	-5.488082	0.861949	1.818474
H	-4.220040	-0.223470	-0.113377
H	-5.993959	-0.330417	-0.202260
H	-5.046088	-1.748995	0.260569
H	-4.445448	2.028227	5.080528
H	-5.445706	1.960698	3.625944
H	-3.694493	2.246399	3.484938
H	-2.033562	-3.136919	3.278807
H	-2.445653	-1.972104	4.543672
H	-1.514505	-1.442824	3.133013

catalyzed_Product$_{retro\text{-}DA}$_*exo* (in chloroform)
E = -5780.99
H = -5537.52
G = -5584.42
N_{imag} = 0

O	-0.464035	-1.991961	-0.050192
N	1.462782	-0.831074	-0.586916
O	3.034923	0.813581	-1.059936
C	0.114690	-0.932231	-0.235718
C	-0.463756	0.500864	-0.174122
C	0.794719	1.414383	-0.204042
C	1.915029	0.484764	-0.688429
C	2.298219	-2.005932	-0.846528
H	-1.043204	0.622867	-1.098203
H	0.701838	2.234369	-0.928246
H	1.866442	-2.596419	-1.659464
H	3.292142	-1.653552	-1.124659
H	2.355326	-2.623743	0.054143
H	-2.471482	-2.389448	4.454585
H	-1.962861	-1.430561	3.050173
C	0.357575	1.856276	2.215467
C	-0.976122	1.279922	2.137448
C	1.189607	1.954380	1.159178
C	-1.406364	0.678036	0.998343
H	-2.279066	-0.640351	4.607782
H	0.671984	2.263669	3.175552
O	-2.625555	0.157511	0.765726
H	-1.631711	1.367109	2.996323
H	2.164066	2.428668	1.236283
H	-3.249582	0.141050	1.604776
N	-4.389531	-0.047487	2.767477
C	-4.391290	1.134268	3.680006
C	-4.082923	-1.294737	3.533830
C	-5.710295	-0.175255	2.077121
H	-3.485836	1.073485	4.290318
H	-5.247057	1.061138	4.373862
C	-4.411058	2.483683	2.952328
C	-2.610376	-1.435750	3.932452
H	-4.351828	-2.141748	2.898459
H	-4.731713	-1.340547	4.425888
H	-6.476421	-0.478819	2.812126
C	-5.700335	-1.139345	0.885258
H	-5.987238	0.818510	1.717457
H	-4.948441	-0.842514	0.147336
H	-6.684560	-1.120230	0.403101
H	-5.500144	-2.173748	1.182591
H	-4.290131	3.288605	3.686305
H	-5.352683	2.659993	2.423496
H	-3.590905	2.556590	2.230474
